# Glycosaminoglycan Domain Mapping of Cellular Chondroitin/Dermatan Sulfates

**DOI:** 10.1038/s41598-020-60526-0

**Published:** 2020-02-26

**Authors:** Andrea Persson, Egor Vorontsov, Göran Larson, Jonas Nilsson

**Affiliations:** 1 Department of Laboratory Medicine, Sahlgrenska Academy at the University of Gothenburg, Gothenburg, Sweden; 2 Proteomics Core Facility, Sahlgrenska Academy at the University of Gothenburg, Gothenburg, Sweden; 3 Laboratory of Clinical Chemistry, Sahlgrenska University Hospital, Gothenburg, Sweden

**Keywords:** Chemical biology, Carbohydrates, Glycoconjugates

## Abstract

Glycosaminoglycans (GAGs) are polysaccharides produced by most mammalian cells and involved in a variety of biological processes. However, due to the size and complexity of GAGs, detailed knowledge about the structure and expression of GAGs by cells, the glycosaminoglycome, is lacking. Here we report a straightforward and versatile approach for structural domain mapping of complex mixtures of GAGs, GAGDoMa. The approach is based on orthogonal enzymatic depolymerization of the GAGs to generate internal, terminating, and initiating domains, and nanoflow reversed-phase ion-pairing chromatography with negative mode higher-energy collision dissociation (HCD) tandem mass spectrometry (MS/MS) for structural characterization of the individual domains. GAGDoMa provides a detailed structural insight into the glycosaminoglycome, and offers an important tool for deciphering the complexity of GAGs in cellular physiology and pathology.

## Introduction

Glycosylation is one of the most prevalent post-translational modifications of proteins and adds an immense and dynamic diversity to the proteome. Proteoglycans are one type of glycosylated proteins comprising one or more glycosaminoglycans (GAGs), which are extensively sulfated polysaccharides commonly composed of 25–100 repeating disaccharide units. GAGs are essential in cellular physiology and pathology as crucial components in the extracellular matrix organization, cell signaling, and cell adhesion^1–5^. Despite recent advances in analytical tools to study GAG structure-function relationships^6,7^, profound knowledge about GAG structure and expression, the GAGome, is lacking. To date, mass spectrometry (MS) is the method of choice for structural characterization of GAGs, and during the last two decades, many MS-based technologies have emerged^8–10^, enabling analysis of disaccharides and shorter oligosaccharides^11–13^, but also studies of solitary complete GAG chains of single proteoglycans^14,15^. Yet, there is a need for accessible, robust, and sensitive MS technologies aiming at global GAGomics with the capacity to successfully characterize the complete set of GAGs expressed by cells or tissues.

**Figure 1:**
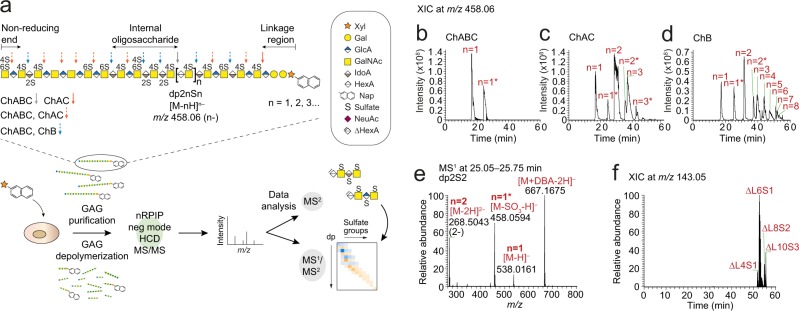
Overview of GAGDoMa. (**a**) Workflow of GAGDoMa and structure of a xyloside-primed CS/DS chain including the cleavage sites of the bacterial depolymerizing enzymes; chondroitinase ABC (ChABC), chondroitinase AC (ChAC), and chondroitinase B (ChB). (**b**–**d)** Extracted ion chromatograms (XICs) at *m/z* 458.06 (dp2nSn, where n = 1, 2, 3…) after chondroitinase ABC (**b**), chondroitinase AC (**c**), and chondroitinase B (**d**) to demonstrate the separation of internal oligosaccharides using GAGDoMa. Additionally sulfated precursor ions, dp2nS(n + 1) (where n = 1*, 2*, 3*…), detected at *m/z* 458.06 owing to in-source sulfate loss, are indicated by an asterisk. (**e**) MS^1^ spectra at 25.05–25.75 min displaying the dp2S2-related precursor ions including [M − 2H]^2-^ at *m/z* 268.50, [M − SO_3_-H]^-^ at *m/z* 458.06, [M − H]^-^ at *m/z* 538.02, and [M+DBA-2H]^-^ at *m/z* 667.17. DBA, dibutylamine. (**f**) XIC at *m/z* 143.05, arising from the naphthyl fragment ion in xyloside-primed linkage region variants generated after chondroitinase B depolymerization displays the elution profile of linkage region variants using GAGDoMa.

The polydisperse and heterogeneous nature of GAGs, in combination with a non-template driven and not yet fully understood biosynthesis, makes the GAGs particularly challenging to study. In humans, the biosynthesis of the GAG subclass chondroitin/dermatan sulfate (CS/DS) is the combined act of at least 22 enzymes including glycosyltransferases, epimerases, and sulfotransferases^6,16,17^. The glycan backbone of CS/DS is composed of repeating disaccharide units of glucuronic acid in β3-linkage to *N*-acetylgalactosamine (GlcAβ3GalNAc), CS motifs, or iduronic acid in α3-linkage to GalNAc (IdoAα3GalNAc), DS motifs. CS/DS can undergo *O*-sulfation at position 2 of the GlcA and IdoA residues and at positions 4 and 6 of the GalNAc residues (Fig. 1a). As an example of the structural diversity of CS/DS, a typical chain of 50 disaccharides have 16^50^ theoretical variants assuming that there are 16 possible disaccharide variants. In addition to the structural complexity of GAGs, most cells produce relatively small amounts of GAGs, on average <0.2 μg CS/DS/heparan sulfate GAGs per 10^6^ cells^18,19^, which challenges detailed GAGomics analyses even further. One way to circumvent this is by the administration of β-D-xylopyranoside primers, or xylosides, to living cells^20^. Since the conventional CS/DS biosynthesis is initiated by the transfer of a xylose residue to a serine residue of the protein, the xylosides, comprising a xylose residue and an aglycon, can highjack the GAG biosynthetic machinery and result in the production and secretion of xyloside-primed GAGs upon administration to living cells, not only amplifying the GAG production several-fold but also displaying the enzymatic capacity of a certain cell by acquiring a structure that largely resembles that of the GAGs produced on proteoglycans by the same cell^19^.

Here, we present a straightforward, effective, and versatile LC-MS/MS technology for structural domain mapping of complex mixtures of GAGs, GAGDoMa (Fig. 1a). The technology is based on orthogonal depolymerization of the CS/DS using primarily two bacterial lyases, chondroitinase AC and chondroitinase B, which specifically cleaves between GalNAc and GlcA residues and GalNAc and IdoA residues, respectively, allowing distinction between IdoA-containing structures, DS motifs, and GlcA-containing structures, CS motifs, as well as internal, terminal, and linkage region domain structures. The MS analysis was performed by nanoflow reversed-phase dibutylamine ion-pairing chromatography with negative mode higher-energy collision dissociation (HCD) MS/MS, enabling fragment ion characterization, compositional profiling and semi-quantification of precursor ions. To challenge GAGDoMa, CS/DS primed on xylosides from two types of human cell lines were used as models as they were predicted to cover a wide range of structures^21,22^. Using this technology, we have identified over 200 structures and characterized more than 60 of these in depth. The overview of domain structures combined with the pinpointing of different modifications provide a deep structural insight into the global GAGome.

## Results

### Strategy for GAGDoMa

To depolymerize CS/DS, there are several bacterial lyases available^23^: chondroitinase ABC depolymerizes all CS/DS into disaccharides (degree of polymerization, dp2) and hexasaccharide linkage region structures (ΔL6), whereas chondroitinase AC (AC-I and AC-II) and chondroitinase B cleave CS/DS specifically at GlcA and IdoA residues, respectively. This generates oligosaccharides of various lengths depending on the distribution of GlcA and IdoA within a co-polymeric CS/DS chain. In addition, heparinases depolymerize heparan sulfate, another class of GAGs, but leaves the CS/DS intact. The enzymes act by elimination, which generates 4,5-unsaturated hexuronic acid residues (ΔHexA; Fig. 1a, boxed legend) that are distinguishable from terminal non-reducing end (NRE) HexA residues (delta mass of 18.0106 u).

For preparation of the CS/DS used for GAGDoMa, we treated human breast fibroblasts, CCD-1095Sk, and human breast carcinoma cells, HCC70, with 100 μM of the xyloside 2-naphthyl β-D-xylopyranoside and the xyloside-primed GAGs were isolated from the media and depolymerized using the different bacterial enzymes (Fig. 1a)^22^. Thereafter, we subjected the samples to nanoflow reversed-phase ion-pairing (RPIP) chromatography using an in-house packed C18 column and dibutylamine (DBA) as an ion-pairing agent. RPIP was selected due to its high chromatographic resolution potential^21,24–26^, and DBA was selected since it is relatively volatile, tends to result in shorter retention times and reduces the number of overlapping charge states compared to other ion-pairing agents^24^. The chromatographic system was directly coupled to an LTQ Orbitrap Elite mass spectrometer operating in negative ionization mode. For the fragmentation analysis, we routinely applied HCD sequentially on precursor ions at normalized collision energies (NCEs) of 60%, 70%, and 80%. Based on previous data on glycopeptide fragmentation at various collision energies^27,28^, we argued that the different NCEs would impact the fragmentation pattern of the oligosaccharides and aid the data interpretation, and therefore, in certain cases additional NCEs of 20–60% were applied. For initial evaluation of the approach, we used disaccharide standards and compared the results to our recently reported microscale LC-MS/MS setup (Fig. S1)^21^. GAGDoMa facilitated a ~300 times more sensitive detection of precursor ions than the previous approach, was more robust in terms of precursor ion intensity (Fig. S1c), and improved the chromatographic separation efficiency (Fig. S1d).

Xyloside-primed CS/DS from CCD-1095Sk cells and HCC70 cells carry essentially one sulfate group per disaccharide^22^. The precursor ions at *m/z* 458.06 (n-), corresponding to internal oligosaccharides carrying one sulfate group per disaccharide, or dp2nSn (where n = 1, 2, 3…), were consequently recurring and enabled straightforward detection of oligosaccharides with increasing length and number of sulfate groups. To further evaluate the chromatographic separation efficiency of GAGDoMa, we studied the extracted ion chromatograms at *m/z* 458.06 of the enzymatically depolymerized xyloside-primed CS/DS, which displayed detection and separation of internal oligosaccharides, dp2nSn (where n = 1, 2, 3…), ranging from dp2S1 to dp16S8 (Fig. 1b–d). By using the precursor ions at *m/z* 458.06, we also detected additionally sulfated oligosaccharides, dp2nS(n + 1) (where n = 1*, 2*, 3*…), which separated chromatographically from the dp2nSn structures at *m/z* 458.06 and appeared due to in-source sulfate loss (Fig. 1b–d**)**. For example, the dp2S2 precursor ions appeared as [M − H]^-^ and [M − 2H]^2–^ precursor ions at *m/z* 538.02 and *m/z* 268.50, respectively, and as the DBA adduct at *m/z* 667.17, but also as [M − SO_3_-H]^-^ at *m/z* 458.06 (Fig. 1e). The linkage region structures (ΔL), containing the naphthyl (Nap) aglycon, separated well from the internal oligosaccharides facilitating their identification already at the MS^1^ level (compare Fig. 1f with Fig. 1b–d). We have previously shown compositional profiling at the MS^1^ level of intact (non-depolymerized) CS/DS up to L19S7^21^. Using GAGDoMa, we demonstrated extensive compositional profiling of intact CS/DS ranging from L11S4 to L29S14 with mass accuracies <10 ppm (Fig. S2 and Table S1).

### Structural characterization of internal oligosaccharides

**Figure 2:**
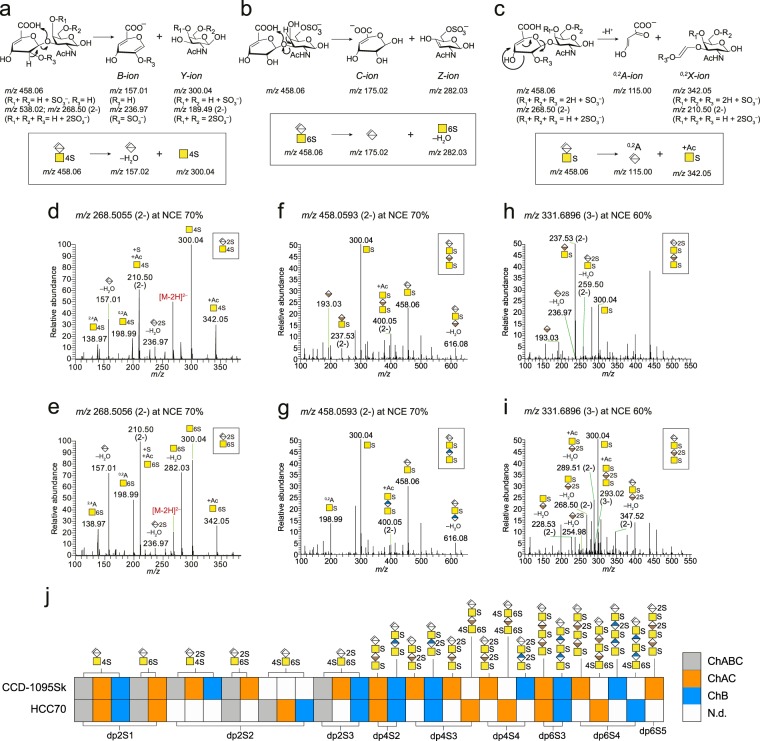
Structural characterization of internal oligosaccharides. (**a**–**c)** Proposed fragmentation reactions into commonly occurring fragment ions and their translations into the Domon and Costello nomenclature for oligosaccharide fragmentation^29^. (**d**–**i**) MS^2^ spectra of internal di- and tetrasaccharides; dp2S2 at *m/z* 268.51 (2-) (**d**,**e**), dp4S2 at *m/z* 458.06 (2-) (**f**,**g**), and dp4S3 at *m/z* 331.69 (3-) (**h**,**i**). (**j**) Internal oligosaccharide profiles of xyloside-primed CS/DS from CCD-1095Sk cells and HCC70 cells after enzymatic depolymerizations. Spectra of included structures, annotations of all fragment ions, and related mass accuracies are found in Figs. S3–S5 and Table S3. N.d., not detected.

To structurally characterize the complex CS/DS mixtures, we started by comparing the MS^2^ spectra of the enzymatically generated internal disaccharides with the MS^2^ spectra of unsaturated disaccharide standards (Fig. S3) and continued with the oligosaccharide isomers of increasing length by comparing the MS^2^ spectra of chromatographically separated precursor ions (Fig. S5). Several fragment ions were recurring in the MS^2^ spectra for the internal oligosaccharides. To understand their origin and facilitate the spectral annotation, we propose fragmentation reactions for the monosulfated and disulfated disaccharide (dp2S1 and dp2S2) precursor ions into B-ions and/or Y-ions where H_2_O is retained on the Y-ion (Fig. 2a) and into C-ions and/or Z-ions where H_2_O is retained on the C-ion (Fig. 2b)^29^. For simplicity, we used standardized glycan symbols^30^ to depict the annotations (Fig. 2a–c, boxed pathways). The ion at *m/z* 342.05, corresponding to a monosulfated GalNAc residue plus the mass of an acetyl group (Ac; 42.0106 u), probably arose due to ^0,*2*^*X* cross-ring cleavage of the ΔHexA residue^31^. We suggest that the fragmentation occurs via a retro-Diels Alder reaction facilitated by the C4-C5 double bond of the ΔHexA residue (Fig. 2c). Additional fragmentation pathways, explaining the cross-ring fragment ions at *m/z* 198.99^32^ and *m/z* 138.97, are included in Fig. S6.

Negative mode MS/MS of the two dp2S1 isomers have previously been described in detail^31,33^, showing that the fragment ions at *m/z* 282.03 and *m/z* 300.04 are diagnostic for ΔHexA-GalNAc6S and ΔHexA-GalNAc4S, respectively. However, the dp2S2 isomers required additional attention. The MS^2^ spectra of the ΔHexA2S-GalNAc4S (Fig. 2d) and ΔHexA2S-GalNAc6S (Fig. 2e) precursor ions at *m/z* 268.50 (2-) showed peaks at *m/z* 300.04 and *m/z* 342.05 pinpointing one sulfate group to the GalNAc residue, but also a peak at *m/z* 236.97 pinpointing the other sulfate group to the ΔHexA residue. In line with the dp2S1 isomers, the intensity of the fragment ion at *m/z* 282.03 was higher for ΔHexA2S-GalNAc6S than for ΔHexA2S-GalNAc4S (Fig. 2d,e). In addition, ΔHexA2S-GalNAc6S showed a more intense ion at *m/z* 157.01. The precursor ion at *m/z* 268.50 (2-) of the third dp2S2 isomer, ΔHexA-GalNAc4S6S (Fig. S3e–g), displayed a diagnostic ion *m/z* 189.49 (2-) pinpointing the two sulfate groups to the GalNAc residue, and consequently lacked a fragment ion at *m/z* 236.97. By comparing the singly and double-charged precursor ions, we concluded that the precursor ions at higher charge state provided better fragmentation at lower NCEs, whereas precursor ions at lower charge state yielded better fragmentation at higher NCEs.

Next, we turned our attention to the internal tetrasaccharides. These are typically generated after chondroitinase AC depolymerization when a single IdoA-GalNAc disaccharide is flanked by two GlcA-GalNAc disaccharides and after chondroitinase B depolymerization when a single GlcA-GalNAc disaccharide is flanked by two IdoA-GalNAc disaccharides. The two dp4S2 isomers at *m/z* 458.06 (2-) generated after chondroitinase AC and B depolymerizations displayed similar MS^2^ spectra including the fragment ions at *m/z* 300.04, *m/z* 400.05 (2-), the latter corresponding to GalNAcS-HexA-GalNAcS(+Ac), and *m/z* 616.08, corresponding to ΔHexA-GalNAcS-HexA(–H_2_O) (Fig. 2f,g), pinpointing one sulfate group to each GalNAc residue. Using sodium Na^+^/H^+^ exchange CID fragmentation^34^, ^*0*,*2*^*X*-ions such as *m/z* 342.05, *m/z* 400.05 (2-), and *m/z* 500.07 appeared more intense for GlcA isomers than for IdoA isomers (Figs. 2f,g and S5). Using HCD, instead, the fragment ion at *m/z* 198.99, corresponding to ^*0*,*2*^*A* cross-ring cleavage of GalNAcS, was more pronounced for the GlcA isomer, whereas fragment ions at *m/z* 193.03 and *m/z* 237.53 (2-) corresponding to HexA and HexA-GalNAcS, respectively, were observed for the IdoA isomer. The additionally sulfated dp4S3 structures displayed similar fragment ions pinpointing one sulfate group to each GalNAc residue, and fragment ions that enabled pinpointing of the third sulfate group. For example, we detected three dp4S3 isomers at *m/z* 331.69 (3-), of which fragment ions at *m/z* 236.97 and *m/z* 259.50 (2-) pinpointed the third sulfate group to the ΔHexA residue (Fig. 2h), fragment ions at *m/z* 254.98, *m/z* 268.50 (2-), and *m/z* 289.51 (2-) pinpointed the third sulfate group to the internal HexA residue (Fig. 2i), and a fragment ion at *m/z* 189.49 (2-) pinpointed the third sulfate group to the reducing end GalNAc residue (Fig. S5j).

Using GAGDoMa and these principles for fragmentation analysis, we characterized internal dp2S1–dp6S5 oligosaccharides from CCD-1095Sk cells and HCC70 cells generated after chondroitinase AC and B depolymerizations (Fig. 2j). Both dp2S1 isomers were detected in the CS/DS from both cell lines, which is in accordance with previous data^21,22^. Of the dp2S2 disaccharides, ΔHexA2S-GalNAc4S appeared predominantly in the CS/DS from CCD-1095Sk cells, whereas ΔHexA-GalNAc4S6S appeared predominantly in the CS/DS from HCC70 cells, and ΔHexA2S-GalNAc6S appeared equally from both cell lines. Additionally, we discovered the rarely described dp2S3 disaccharide after both chondroitinase AC and B depolymerizations from CCD-1095Sk cells and after chondroitinase B depolymerization from HCC70 cells indicating that parts of the CS/DS chains were highly sulfated (Figs. 2j and S3), and that the lyases were capable of cleaving such parts. The dp4S2 and dp6S3 structures were detected after both chondroitinase AC and B depolymerizations from both cell lines. Further sulfation into dp4S3, dp4S4, dp6S4, and dp6S5 structures, showed that the CCD-1095Sk cells mainly included sulfation of HexA/ΔHexA residues, whereas, for the HCC70 cells, additional sulfation took place mainly on GalNAc residues (Fig. 2j). Taken together, the fragmentation patterns of the internal oligosaccharides contained important information regarding sulfate modifications and IdoA/GlcA isomers (Figs. 2j, S3 and S5, and Table S2), clearly demonstrating that GAGDoMa provided evidence of structural differences between the CS/DS chains derived from the two cell lines.

### Structural characterization of terminal non-reducing ends

The general knowledge about the terminal ends of GAGs is limited since their analysis is usually not available when pursuing disaccharide analysis of GAGs. The NRE precursor ions had an additional mass of 18.0106 u compared to the internal oligosaccharides, and separated well chromatographically based on the number of monosaccharides and sulfate groups, but also on the isomeric level (Fig. S7). In contrast to the internal oligosaccharides, the NRE displayed little or no fragment ions generated by ^0,*2*^*X* cleavage (Fig. S7). This implies that ^0,*2*^*X*-ions arise primarily from ΔHexA-containing structures, that is, oligosaccharides obtained after the enzymatic depolymerization, which is also further supported by the proposed ^0,*2*^*X* cleavage mechanism (Fig. 2c).

**Figure 3:**
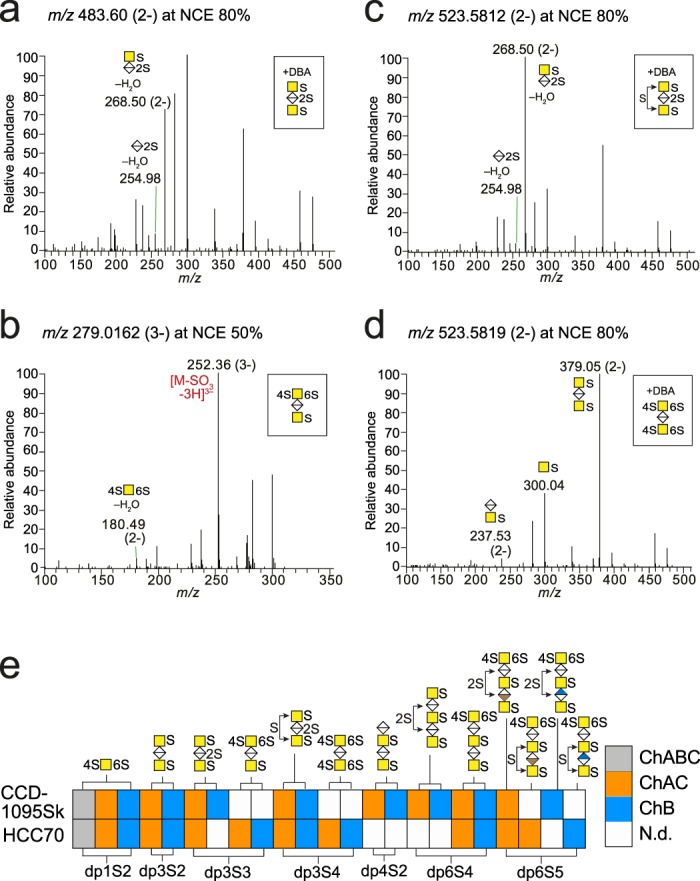
Structural characterization of terminal non-reducing ends. (**a**–**d**) MS^2^ spectra of terminal non-reducing end (NRE) trisaccharides; dp3S3 at *m/z* 483.60 (2-) (**a**) and at *m/z* 279.02 (3-) (**b**), and dp3S4 at *m/z* 523.58 (2-) (**c**,**d**). (**e**) Terminal NRE profiles of xyloside-primed CS/DS from CCD-1095Sk cells and HCC70 cells after enzymatic depolymerizations. Spectra of included structures, annotations of all fragment ions, and related mass accuracies are found in Fig. S7 and Table S3. N.d., not detected.

In addition to the mono- and disaccharide NREs previously reported^21^, we detected trisaccharides carrying more than one sulfate group per GalNAc residue; dp3S3 and dp3S4 (Fig. 3). The enzyme specificity towards these highly sulfated terminal structures is not known, and therefore, we omitted the isomeric structure of the first HexA of the NREs from our annotations. One of the dp3S3 isomers appeared as a precursor ion at *m/z* 483.60 (2-) (Fig. 3a) and displayed a fragment ion at *m/z* 254.98, which pinpointed the additional sulfate group to the HexA residue. A second dp3S3 isomer appeared as a precursor ion at *m/z* 279.03 (3-) (Fig. 3b), and showed a diagnostic ion at *m/z* 180.49 (2-) indicating additional sulfation of the terminal GalNAc residue rather than of the reducing end GalNAc (compare to *m/z* 189.49 (2-) for disulfation of the reducing end GalNAc residue) (Fig. S5j). The two dp3S4 isomers appeared as precursor ions at *m/z* 523.58 (2-) (Fig. 3c,d). Similarly to the dp3S3 isomer at *m/z* 483.60 (2-), one of the isomers displayed fragment ions at *m/z* 254.98 and *m/z* 268.50 (2-), pinpointing one of the sulfate groups to the HexA (Fig. 3c**)**. The dp3S4 precursor ion was doubly charged despite carrying four sulfate groups, thus, it lacked fragment ions that pinpointed to which GalNAc residue the additional sulfate group was attached. The second dp3S4 isomer, lacked fragment ions at *m/z* 254.98 and *m/z* 268.50 (2-) implying that it was disulfated on both GalNAc residues (Fig. 3d).

Altogether, GAGDoMa allowed for characterization of a variety of terminal NRE structures after chondroitinase AC and B depolymerizations, ranging from dp1S2 to dp5S5 (Fig. 3e). The dp1S2 variant was present in all samples from both cell lines (Fig. 3e) indicating that disulfation of the terminal GalNAc residue is a common motif in CS/DS GAGs from these cells. In the NREs from HCC70 cells, this motif was particularly prominent as it was detected also in the longer structures, as indicated by the fragment ion at *m/z* 180.49 (2-) (Fig. S7). Sulfation of HexA was observed in NRE variants from both cell lines, for instance, in the different glycoforms of dp5S4 and dp5S5 (Fig. 3e). To summarize, the terminal domains of the studied GAGs frequently carried more than one sulfate group per GalNAc residue.

### Structural characterization of linkage regions

CS/DS is generally polymerized from a linkage region tetrasaccharide, GlcAβ3Galβ3Galβ4Xyl-*O*-Ser, which can undergo different modifications including sulfation and sialylation of the Gal residues^21,27,35^. Depending on the orchestration of GlcA and IdoA within the CS/DS chain, linkage region variants of different lengths are formed upon chondroitinase AC and B depolymerizations. Despite having a different monosaccharide composition than the internal oligosaccharides, the principles for annotation of linkage region fragment ions was similar; pinpointing of sulfate groups and *N*-acetylneuraminic acid (Neu5Ac) were based on diagnostic ions and fragment ions of different intensities (fragment ions had an accuracy of <10 ppm; Tables S2 and S3). Analogously, the HCD generated mainly glycosidic fragmentation, but also ^0,*2*^*X* cross-ring cleavage of the ΔHexA-terminated structures.

**Figure 4:**
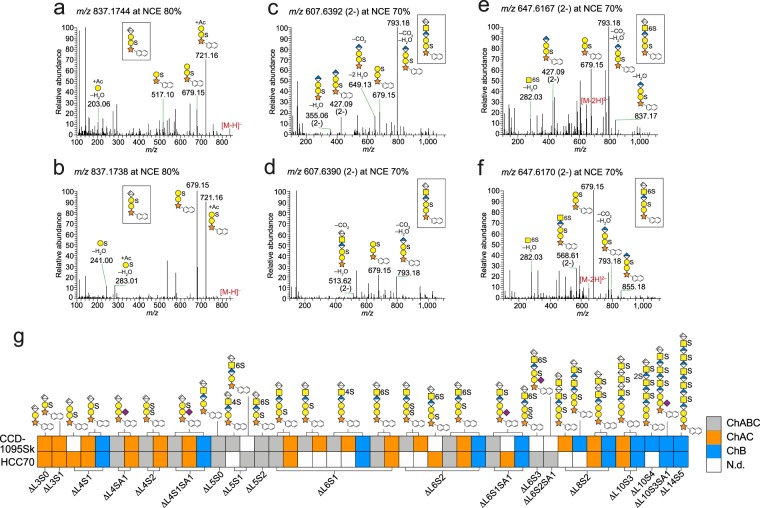
Structural characterization of linkage regions. (**a**–**f**) MS^2^ spectra of linkage region tetra- and hexasaccharides; ΔL4S1 at *m/z* 837.17 (**a**,**b**), ΔL6S1 at *m/z* 607.64 (2-) (**c**,**d**), ΔL6S2 at *m/z* 647.62 (2-) (**e**,**f**). (**g**) Linkage region profiles of xyloside-primed CS/DS from CCD-1095Sk cells and HCC70 cells after enzymatic depolymerizations. Spectra of included structures, annotations of all fragment ions, and related mass accuracies are found in Figs. S8–S13 and Table S3. N.d., not detected.

For pinpointing of the sulfate groups to the first or second Gal residue from the reducing end, we compared the fragmentation patterns of chromatographically separated isomers (Fig. S9). ΔL4S1 had two isomers at *m/z* 837.17 (Fig. 4a,b); one with fragments ion at *m/z* 517.10, and *m/z* 203.06, corresponding to GalS-Xyl-*O*-Nap and Gal(+Ac–H_2_O), respectively, which pinpointed the sulfate group to the first Gal residue from the reducing end. Consistently, the other isomer had a fragment ion at *m/z* 283.01, corresponding to GalS(+Ac–H_2_O), which pinpointed the sulfate group to the second Gal residue. In addition, there was a shift in intensity of the fragment ions at *m/z* 679.15 and 721.16, corresponding to Gal-Gal-Xyl-*O*-Nap(+SO_3_) and Gal-Gal-Xyl-*O*-Nap(+Ac+SO_3_), respectively. When the number of monosaccharides or modifications increased, the intensities decreased of the fragment ions at *m/z* 517.10 and *m/z* 283.01, whereas the intensities increased of the fragment ions at *m/z* 679.15 and *m/z* 793.18, the latter corresponding to HexA-Gal-Gal-Xyl-*O*-Nap(+SO_3_–H_2_O–CO_2_). The intensities of these ions were diagnostic for pinpointing sulfate to either of the Gal residues since *m/z* 793.18 was dominating for sulfation of the first Gal residue from the reducing end, and *m/z* 679.15 was dominating for sulfation of the second Gal residue (Fig. 4c–f). Sulfate group pinpointing on the first GalNAc residue of ΔL6 variants was performed based on the same principles as for the internal disaccharides; a dominating fragment ion at *m/z* 282.03 was significant for 6S-*O*-sulfation and a dominating fragment ion at *m/z* 300.04 was significant for 4S-*O*-sulfation (Fig. S9).

GAGDoMa combined with these basic principles for annotation allowed for characterization of 28 different linkage region structures (Fig. 4g), including variants of the non-canonical trisaccharide linkage region that we recently reported^36^ (Fig. S10), variants containing Neu5Ac^21,27^ (Fig. S11), variants where both Gal residues were sulfated (Fig. S12), and various extended structures (Fig. S13). The trisaccharide linkage region variants included ΔL3S0 and ΔL3S1 after chondroitinase AC depolymerization, and ΔL5S0, ΔL5S1 and ΔL5S2 after chondroitinase ABC depolymerization (Fig. S10 and Tables S2 and S3), and appeared for both CCD-1095Sk cells and HCC70 cells. Neu5Ac was pinpointed to the first Gal residue from the reducing end of ΔL4SA1 (SA, sialic acid), since the MS^2^ spectrum of the precursor ion at *m/z* 523.6522 (2-) displayed a diagnostic ion at *m/z* 728.24 corresponding to Neu5Ac-Gal-Xyl-*O*-Nap (Fig. S11). The position was in agreement with previous glycoproteomics data for proteoglycan samples^27,35^. Despite the weak intensity of *m/z* 728.24 for Neu5Ac-containing structures of increasing length (≥ΔL6) or modified with one or more sulfate groups, the fragmentation patterns of those structures gave no reason to suspect that Neu5Ac would be positioned differently (Fig. S11). With the exception of ΔL6S2SA1, which was only found in HCC70 cells, all Neu5Ac-containing variants appeared in both cell lines. The series of linkage region variants carrying sulfate groups on both of the Gal residues (Fig. S12) all displayed diagnostic ions at *m/z* 307.02 (2-) and *m/z* 379.05 (2-), corresponding to GalS-GalS-Xyl(–H_2_O) and GalS-GalS-Xyl-*O*-Nap, respectively. The ΔL6S2 variant appeared amongst the structures from CCD-1095Sk cells only, whereas the additionally sulfated ΔL6S3 variant appeared amongst the structures from HCC70 cells only (Fig. 4g). The extended linkage region structures contained fragment ions observed both for the internal structures and the linkage region hexasaccharides (Fig. S13). Interestingly, IdoA and GlcA isomers displayed different fragmentation patterns; the presence of IdoA resulted primarily in C- and Y-ions, whereas the presence of GlcA resulted primarily in B- and Z-ions (Fig. S13c–j).

Several of the linkage region structures were only expressed by one cell line (Fig. 4g). For example, sulfation of the first Gal residue from the reducing end appeared mainly in the linkage region variants from HCC70 cells, whereas 4S-*O*-sulfation of the first GalNAc residue from the reducing end was mainly observed in linkage region variants from CCD-1095Sk cells. As expected, linkage region tetrasaccharides were primarily observed after chondroitinase AC depolymerization, hexasaccharides after chondroitinase ABC and B depolymerizations, and extended structures after chondroitinase B depolymerization (Fig. 4g). However, some products deviated from this norm indicating that the enzymes are not solely restricted to their predicted specificities.

### Structural overview of domains of CS/DS primed on xylosides

**Figure 5:**
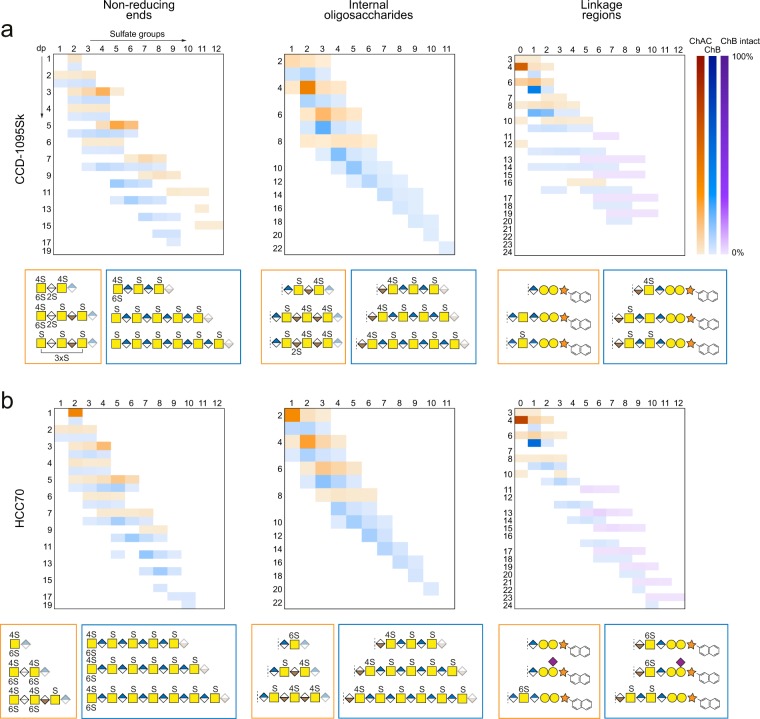
Structural summary of GAG domains. Heat map representations of non-reducing end, internal oligosaccharide, and linkage region domain structures after chondroitinase AC (ChAC, orange) and chondroitinase B (ChB, blue) in CS/DS primed on xylosides from CCD-1095Sk cells (**a**) and HCC70 cells (**b**). For linkage regions, intact GAGs after chondroitinase B depolymerization were included (ChB intact, purple), and calculated as the percentage of the total linkage region variants after chondroitinase B depolymerization. The intensity of the different structures was based on the area under the curve of the precursor ion peaks. Based on the heat maps and structure profiles (Figs. 2–4), the three most common structures from CCD-1095Sk cells (**a**) and HCC70 cells (**b**) after chondroitinase AC (orange) and B (blue) depolymerizations of each domain are displayed in the boxes at the bottom of each heat map panel. Sulfate positioning was based on MS^2^ data, and for the internal oligosaccharides, the sulfate groups of the GalNAc residues on the reducing end side of IdoA residues were designated position 4 based on the specificities of biosynthetic enzymes^38,53^. Stereochemistry of the HexA residues (GlcA/IdoA) on the non-reducing (dashed line) and reducing (semi-opaque symbol) end sides was interpreted by the specificities of the depolymerizing enzymes. The data in **a** and **b** are each from one representative sample. Raw data are found in Table S4. dp, degree of polymerization.

To obtain an overview of the xyloside-primed CS/DS from the two cell lines, we mapped the structures observed within the three domains after enzymatic depolymerization (Fig. 5); internal oligosaccharides (dp2S1 to dp22S11), NREs (dp1S2 to dp19S10), and linkage regions (ΔL3S0 to ΔL24 S10, and L11S5 to L23S12). We identified over 150 structures, and by using the intensity of each precursor ion, we obtained a semi-quantitative estimation of all the detected structures after each depolymerization within each domain. To entwine the domain mapping and structural profiles (Figs. 2–4), we summarized the three most common structures within each domain after the chondroitinase AC and B depolymerizations (Fig. 5), thereby, clearly showing differences in lengths and sulfation patterns of the oligosaccharides generated after the depolymerizations of the CS/DS from the two cell lines.

The internal oligosaccharides had, on average, one sulfate group per disaccharide, whereas the NREs were more sulfated and the linkage regions less sulfated. Chondroitinase B depolymerization resulted in internal saccharides of dp2–dp20/22 from both cell lines and chondroitinase AC depolymerization resulted in internal saccharides of dp2–dp8 from both cell lines, the latter corresponding to up to three consecutive IdoA residues. This implies that a hypothetical average internal domain of dp60, as previously estimated^22^, is a heterogeneous co-polymeric structure comprising both CS and DS motifs of different lengths where the CS motifs, on average, are longer than the DS motifs. Whether this is a consequence of the specificity of the epimerases^37–39^, substrate availability, or both, remains to be elucidated. In addition, several intact GAGs remained after chondroitinase B depolymerization, confirming previous speculations that a subgroup of the GAG chains are entirely of CS character^21^. The observed differences in length of the CS and DS motifs and the presence of a CS GAG subgroup imply that CS/DS produced by these cell lines are of highly heterogeneous nature.

The CS/DS GAGs were principally terminated with GalNAc (dp1, dp3, dp5…) of which the majority was disulfated, and only to a small degree with HexA (dp2, dp4, and dp6) (Fig. 5 and Table S4). In addition, the NREs were overall more sulfated than the internal oligosaccharides, and appeared with more than one sulfate group per monosaccharide, such as in dp3S4 and dp5S6. The shorter NRE variants, up to dp6, had a similar sulfation level irrespective of type of depolymerization, yet, with increasing length, the variants generated after chondroitinase B depolymerization were less sulfated than those generated after chondroitinase AC depolymerization implying that the NRE DS motifs were more sulfated than the corresponding CS motifs. Also, the NRE DS motifs were longer than the internal DS motifs suggesting that the DS character of the CS/DS chain was more pronounced towards the NREs. In the linkage region variants, Neu5Ac was observed after both depolymerizations, however, it was much more prevalent in the linkage regions from the HCC70 cells (Fig. S14).

## Discussion

We have developed GAGDoMa, a strategy for structural domain mapping of CS/DS, which allowed for MS^2^-based characterization of complex mixtures of internal oligosaccharides (up to dp6S5), NREs (up to dp5S5), and linkage regions (up to ΔL14S5) obtained after depolymerizations with bacterial enzymes. Furthermore, we demonstrated extensive compositional profiling of oligosaccharide products up to dp22/ΔL24 and intact GAGs up to L29. Compared to the conventional disaccharide analysis^21,22^, GAGDoMa requires the same number of analytical runs, but provides a considerably more comprehensive depiction of the GAGome. For example, the structural information regarding oligosaccharides longer than dp2, NREs, and linkage regions, all covered by GAGDoMa, is lost when performing disaccharide analysis. The concept of domain region analysis of GAGs has previously been suggested^40^, but this was based on disaccharide analysis of heparan sulfate GAGs. The study used computational approaches for the analysis, which should prove useful also for future GAGDoMa projects.

The strategy relies on high-resolution mass spectrometry, but does not require the most recent and expensive instruments; thus, it may be easily implemented in most MS-oriented laboratories. Furthermore, the method does not involve any derivatization or chemical modification of the oligosaccharides.

The choice of developing GAGDoMa in nanoflow LC instead of in microflow LC was advantageous with respect to the sensitivity and sample amount required for detailed structural characterization of GAGs (Fig. S1). In addition, for electrospray ionization (ESI) in nanoflow, there is less optimization required than for ESI in microflow, for example, there is no gas involved in nanospray. RPIP chromatography offers a greater chromatographic resolution capacity than size-exclusion chromatography^41^, which generally is unable to provide any isomeric separation, and while hydrophilic interaction chromatography appears convenient for disaccharides^42^ and oligosaccharides^43^, it remains unexplored for resolving complex mixtures of GAGs with LC-MS/MS. Furthermore, the selected chromatographic system and instrument can also be used for standard peptide-based proteomics. The in-house packed C18 column showed stable performance during several weeks of usage. We and others have previously shown that the ion-pairing agent DBA is suitable for RPIP separation of GAGs or GAG-derived oligosaccharides^21,24,44^. Here, we demonstrated separation of oligosaccharides ranging from dp2 to dp16 and even separation down to isomeric levels (Figs. S5, S8 and S10). The DBA ion-pairing typically resulted in the formation of only up to three precursor ions of different charge states per structure, including various degree of DBA adducts (Table S4), whereas sodium ion-pairing, for example, tends to result in many more^45^. The fewer the adducts, the higher the intensity of each structure and thus the more sensitive and straightforward the analysis. Although we observed some degree of in-source sulfate loss for certain precursor ions, such as for dp2S2 and dp2S3 (Fig. S15), others, such as the dp4S2 precursor ions at *m/z* 458.06 (2-) (Fig. S5a), did not display in-source sulfate loss. Despite some in-source sulfate loss and DBA adduct formation of precursor ions, fragmentation of intact, DBA-lacking, and multiply charged precursors were achieved in almost all cases. Therefore, the method was not further optimized to minimize sulfate loss and DBA adduct formation. Taken together, the nRPIP LC-MS/MS appears highly convenient in terms of chromatographic separation and limited adduct formation, but also for excellent fragmentation characteristics of the multiply charged precursor ions.

Different dissociation techniques tend to generate different types of fragment ions^12^. HCD appears convenient for GAG fragmentation; for example, we obtained more informative fragment ions compared to collision-induced dissociation (CID) (Fig. S16)^46,47^, yet, the spectra obtained were readily interpretable. The high resolution of the Orbitrap detector, set to 30,000 for the MS^2^ scans, gave excellent mass accuracies of the fragment ions and enabled confident identification of their identities. As an example, the full set of the fragment ions in Fig. S5b had an average mass accuracy of –2.0 ppm with a standard deviation of 2.5 ppm (Table S3). In addition, the spectra were highly reproducible between different runs (Fig. S17). To obtain optimal fragmentation, we applied different NCE levels. For sulfated structures, the preferred NCE level was decreased with increasing number of sulfate groups and charge state: NCE at 80% was better for one sulfate group and singly charged precursor ions, NCE at 70% was better for two sulfate groups and doubly charged precursor ions, *et cetera* (Figs. S3 and S14). Critical fragment ions were occasionally more prevalent at an NCE deviating from the one providing the optimal MS^2^ fragmentation, especially for low *m/z* fragment ions that required higher energies. For non-sulfated structures or structures carrying a Neu5Ac residue, lower energies and higher charge states of the precursor ions were beneficial to use. The short and more sulfated NRE structures, such as dp3S4, were difficult to obtain at higher charge states, which may be due to their high sulfate group density. Taken together, the application of different collision energies efficiently promoted the characterization of the wide range of structures appearing in these cells.

We used two human cell lines predicted to produce structurally different CS/DS^22^ to demonstrate the capacity of GAGDoMa, and indeed differences in the CS/DS produced by the two cell lines were confirmed in all three GAG domains obtained after enzymatic depolymerization. In addition, several differently sulfated variants were observed for each oligosaccharide of a specific length, although the CS/DS, on average, carried one sulfate group per disaccharide (Fig. 5). This shows that the GAGomes of both CCD-1095Sk cells and HCC70 cells are highly complex, plausibly enabling various GAG-protein interactions, not the least via the highly sulfated terminal domains. Similarly to a recently reported approach where a GalNAc derivative was used for amplification of the *O*-glycome in living cells^48^, we used xyloside primers to obtain the CS/DS of interest. The use of primers may not completely correspond to the natural situation; however, the amplification clearly facilitates the characterization of less commonly occurring glycan structures^19,48^, and the GAG structures reported herein are likely to be found also in proteoglycan-derived GAGs^27,36,37,49^. For example, decorin and biglycan from human lung fibroblasts are reported to have a large proportion of IdoA in blocks^37^, and more specifically, IdoA in blocks (≤dp15) towards the NRE is reported in decorin from porcine skin^49^. Discounting the possible issue of enrichment, characterization of released proteoglycan-derived GAGs should also be feasible using GAGDoMa. Additional biological tools, such as cell libraries genetically modified to display specific GAG structures^6,7^, and computational tools^40^, could further elaborate on the potential of GAGDoMa. Similarly, the aid of well-defined standards (>dp2), would expand the capacity of GAGDoMa by enabling distinction between, for example, 4S- and 6S-*O*-sulfation in oligosaccharides and improve the quantification. Due to the width of the generated data, we limited this study to CS/DS, the subclass of GAGs primarily formed on xylosides. However, we expect that this strategy can be expanded to the other subclasses of GAGs, provided the relevant depolymerization enzymes are available.

In conclusion, we have developed a strategy for structural domain mapping of GAGs, GAGDoMa, enabling characterization of complex mixtures at a level of molecular detail previously not possible. The strategy is based on enzymatic depolymerization and nLC-MS/MS analysis using reversed-phase dibutylamine ion-pairing chromatography with negative mode HCD MS/MS of the oligosaccharides for identification, characterization, and semi-quantitative analysis. GAGDoMa provides a comprehensive insight into the complexity of the GAGome and will most certainly constitute a fundament for a deeper understanding of structure–function relations of GAGs in physiology and pathology.

## Methods

### Preparation of xyloside-primed glycosaminoglycans for LC-MS/MS

Xyloside-primed GAGs were prepared as previously described^22^. Briefly, CCD-1095Sk cells and HCC70 cells (American Type Culture Collection) were cultured as monolayers according to the manufacturer’s instructions. At 70% confluency, the cells were preincubated in serum-free Dulbecco’s Modified Eagle’s Medium/Nutrient Mixture F-12 Ham medium (Sigma-Aldrich) for 24 h, followed by incubation with fresh medium supplemented with 100 μM of 2-naphthyl β-D-xylopyranoside, synthesized as previously reported^50^. After 48 h, the media were collected and the xyloside-primed GAGs isolated by diethylaminoethyl-Sepharose (GE Healthcare) and octyl-Sepharose (Sigma-Aldrich) chromatography, and then ethanol precipitation. The xyloside-primed GAGs were purified using a Superose 12 HR 10/30 column coupled to a Thermo Scientific Ultimate 3000 Quaternary Analytical System and collected based on fluorescence of the naphthyl aglycon (excitation λ = 229 nm, emission λ = 342 nm). ∼15 μg of xyloside-primed GAGs, as roughly estimated using the 1,9-dimethylmethylene blue method^51^, were used for depolymerization with 100 mU chondroitinase ABC (EC 4.2.2.20) (Seikagaku) or 50 mU of each chondroitinase AC-I and -II (EC 4.2.2.5) (Seikagaku) in 50 mM NH_4_OAc pH 8.0 at 37 °C for 16 h, or 50 mU chondroitinase B (EC 4.2.2.19) (R&D Systems) or heparinase II and III (EC 4.2.2.8) (overexpressed in *E*.*coli*, gift from Prof. Jian Liu) in 50 mM NH_4_OAc, 4 mM CaCl_2_ pH 7.3 at 37 °C for 16 h. Samples were heat-inactivated and lyophilized before analysis using LC-MS/MS.

### nLC-MS/MS setup

Samples were analyzed using an Easy-nLC 1200 LC system (Thermo Fisher Scientific, San Jose, CA) coupled to an LTQ Orbitrap Elite mass spectrometer (Thermo Fisher Scientific). ∼400 ng of depolymerized xyloside-primed GAGs or 200–400 pg of disaccharide standards were used for each analytic run. The analytes were trapped on a 2 cm ×100 µm Acclaim PepMap C18 precolumn (particle size 5 µm; Thermo Fisher Scientific) and separated on a 30 cm ×75 µm analytical column packed in-house with 3 μm Reprosil-Pur C18 material (Dr. Maisch, Germany) at 300 nL/min flow using a stepwise elution profile: from 0 to 30% B in 1 min, 30% B for 9 min, from 30 to 40% B in 1 min, 40% B for 9 min, from 40 to 50% B in 1 min, 50% B for 9 min, from 50 to 60% B in 1 min, 60% B for 9 min, from 60 to 70% B in 1 min, 70% B for 9 min, from 70 to 100% B in 1 min, 100% B for 14 min. Solvent A was 5 mM di-n-butylamine and 8 mM acetic acid in H_2_O, solvent B was 70% methanol, 5 mM di-n-butylamine and 8 mM acetic acid. The standards were analyzed using a shorter elution profile: from 0 to 30% B in 1 min, 30% B for 11 min, from 30 to 60% B in 1 min, 60% B for 11 min, from 60 to 100% B in 1 min, 100% B for 15 min. The column oven temperature was 50 °C. Nano-Flex ion source (Thermo Fisher Scientific) was operated in negative ionization mode at 1.8 kV with the ion transfer capillary temperature 325 °C. Each sample were run at least three times, with slightly different MS/MS selection and fragmentation settings: full scan in the *m/z* range 260–2,000 at 60,000 or 120,000 resolution, followed by the HCD-MS^2^ spectra with NCEs 60%, 70%, and 80%, NCE 80%, or NCEs 20%, 30%, 40%, 50%, and 60%, with and without dynamic exclusion. For all settings, the automatic gain control (AGC) target in the full MS spectra was 10^6^, precursor isolation window was 5 Da and the HCD spectra were recorded at 15,000 resolution with the first *m/z* 100 and an AGC target of 10^5^; precursor ions with unassigned charge states were rejected.

### μLC-MS/MS setup

∼100 ng of disaccharide standards were analyzed using a Thermo Scientific Ultimate 3000 RS chromatography system equipped with an in-house-made flow split and coupled to an LTQ Orbitrap Elite mass spectrometer as previously described^21^. Briefly, the analytes were separated using an Acquity BEH C18 column (300 Å pore size, 1.7 μm particle size, 300 μm × 150 mm column dimensions; Waters) under stepwise isocratic elution at approximately 2 μL/min flow. The following elution profile was used: 100% A-solvent (5 mM di-n-butylamine and 8 mM AcOH in H_2_O) for 13 min, at 30% B-solvent (5 mM di-n-butylamine and 8 mM AcOH in 70% MeOH) for 15 min, then at 60% B for 10 min, and at 100% B for 19 min. The electrospray source was operated in negative ionization mode at 3.5 kV. Precursor ion mass spectra were recorded at 30,000 resolution in the *m/z* range 215–2,000 with the AGC target at 10^6^. The 10 most intense precursor ions were selected with an isolation window of 4.0 *m/z* units without a dynamic exclusion, fragmented using HCD at the NCE of 70% and 80%, and the MS^2^ spectra were recorded at a resolution of 15,000 with the first *m/z* 100 and the AGC target 10^5^; precursor ions with unassigned charge states were rejected.

### Data analysis

Glycomics data were processed using the XCalibur software (Thermo Fisher Scientific) and interpreted manually. For the data presentation, representative chromatograms and spectra were chosen. The precursor masses were given as the monoisotopic masses to four decimal places and the annotated fragment masses were given to two decimal places of the highest intensity isotope peak. The MS data have been deposited to the ProteomeXchange Consortium via the PRIDE partner repository^52^ with the dataset identifiers PXD014504. Glycan symbols were depicted according to the Symbol Nomenclature for Glycans^30^. Graphs were generated using GraphPad Prism version 8.0.1 (GraphPad software).

## Supplementary information

Supplementary information.

Supplementary information2.

## References

[1] Xu D, Esko JD (2014). Demystifying heparan sulfate–protein interactions. Annu. Rev. Biochem..

[2] Mizumoto S, Yamada S, Sugahara K (2015). Molecular interactions between chondroitin–dermatan sulfate and growth factors/receptors/matrix proteins. Curr. Opin. Struct. Biol..

[3] Miller GM, Hsieh-Wilson LC (2015). Sugar-dependent modulation of neuronal development, regeneration, and plasticity by chondroitin sulfate proteoglycans. Exp. Neurol..

[4] Soares da Costa D, Reis RL, Pashkuleva I (2017). Sulfation of glycosaminoglycans and its implications in human health and disorders. Annu. Rev. Biomed. Eng..

[5] Lu P., Takai K., Weaver V. M., Werb Z. (2011). Extracellular Matrix Degradation and Remodeling in Development and Disease. Cold Spring Harbor Perspectives in Biology.

[6] Chen Y-H (2018). The GAGOme: a cell-based library of displayed glycosaminoglycans. Nat. Methods.

[7] Qiu H (2018). A mutant-cell library for systematic analysis of heparan sulfate structure–function relationships. Nat. Methods.

[8] Wei J (2019). Characterization and quantification of highly sulfated glycosaminoglycan isomers by gated-trapped ion mobility spectrometry negative electron transfer dissociation MS/MS. Anal. Chem..

[9] Wolff JJ, Amster IJ, Chi L, Linhardt RJ (2007). Electron detachment dissociation of glycosaminoglycan tetrasaccharides. J. Am. Soc. Mass. Spectrom..

[10] Wolff JJ (2010). Negative electron transfer dissociation of glycosaminoglycans. Anal. Chem..

[11] Volpi N, Galeotti F, Yang B, Linhardt RJ (2014). Analysis of glycosaminoglycan-derived, precolumn, 2-aminoacridone–labeled disaccharides with LC-fluorescence and LC-MS detection. Nat. Protoc..

[12] Kailemia MJ, Ruhaak LR, Lebrilla CB, Amster IJ (2014). Oligosaccharide analysis by mass spectrometry: a review of recent developments. Anal. Chem..

[13] Zaia J (2013). Glycosaminoglycan glycomics using mass spectrometry. Mol. Cell. proteomics: MCP.

[14] Ly M (2011). The proteoglycan bikunin has a defined sequence. Nat. Chem. Biol..

[15] Yu Y (2017). Sequencing the dermatan sulfate chain of decorin. J. Am. Chem. Soc..

[16] Mikami T, Kitagawa H (2013). Biosynthesis and function of chondroitin sulfate. Biochimica et. Biophysica Acta - Gen. Subj..

[17] Koike T, Izumikawa T, Sato B, Kitagawa H (2014). Identification of phosphatase that dephosphorylates xylose in the glycosaminoglycan-protein linkage region of proteoglycans. J. Biol. Chem..

[18] Li G (2015). Glycosaminoglycanomics of cultured cells using a rapid and sensitive LC-MS/MS approach. ACS Chem. Biol..

[19] Persson A, Ellervik U, Mani K (2018). Fine-tuning the structure of glycosaminoglycans in living cells using xylosides. Glycobiol..

[20] Okayama M, Kimata K, Suzuki S (1973). The influence of p-nitrophenyl β-D-xyloside on the synthesis of proteochondroitin sulfate by slices of embryonic chick cartilage. J. Biochem..

[21] Persson A (2018). LC–MS/MS characterization of xyloside-primed glycosaminoglycans with cytotoxic properties reveals structural diversity and novel glycan modifications. J. Biol. Chem..

[22] Persson A (2016). Xyloside-primed chondroitin sulfate/dermatan sulfate from breast carcinoma cells with a defined disaccharide composition has cytotoxic effects *in vitro*. J. Biol. Chem..

[23] Ernst S, Langer R, Cooney CL, Sasisekharan R (1995). Enzymatic degradation of glycosaminogIycans. Crit. Rev. Biochem. Mol. Biol..

[24] Kuberan B (2002). Analysis of heparan sulfate oligosaccharides with ion pair-reverse phase capillary high performance liquid chromatography-microelectrospray ionization Time-of-Flight mass spectrometry. J. Am. Chem. Soc..

[25] Henriksen J, Roepstorff P, Ringborg LH (2006). Ion-pairing reversed-phased chromatography/mass spectrometry of heparin. Carbohydr. Res..

[26] Thanawiroon C, Rice KG, Toida T, Linhardt RJ (2004). Liquid chromatography/mass spectrometry sequencing approach for highly sulfated heparin-derived oligosaccharides. J. Biol. Chem..

[27] Gomez Toledo A, Nilsson J, Noborn F, Sihlbom C, Larson G (2015). Positive mode LC-MS/MS analysis of chondroitin sulfate modified glycopeptides derived from light and heavy chains of the human inter-α-trypsin inhibitor complex. Mol. Cell. Proteom..

[28] Hinneburg H (2016). The art of destruction: optimizing collision energies in quadrupole-Time of Flight (Q-TOF) instruments for glycopeptide-based glycoproteomics. J. Am. Soc. Mass. Spectrom..

[29] Domon B, Costello CE (1988). A systematic nomenclature for carbohydrate fragmentations in FAB-MS/MS spectra of glycoconjugates. Glycoconj. J..

[30] Varki A (2015). Symbol nomenclature for graphical representations of glycans. Glycobiol..

[31] Zaia J, Costello CE (2001). Compositional analysis of glycosaminoglycans by electrospray mass spectrometry. Anal. Chem..

[32] Zaia J, Costello CE (2003). Tandem mass spectrometry of sulfated heparin-like glycosaminoglycan oligosaccharides. Anal. Chem..

[33] Desaire H, Leary JA (2000). Detection and quantification of the sulfated disaccharides in chondroitin sulfate by electrospray tandem mass spectrometry. J. Am. Soc. Mass. Spectrom..

[34] Kailemia MJ (2015). Differentiating chondroitin sulfate glycosaminoglycans using collision-induced dissociation; uronic acid cross-ring diagnostic fragments in a single stage of tandem mass spectrometry. Eur. J. Mass. Spectrom..

[35] Klein JA, Meng L, Zaia J (2018). Deep sequencing of complex proteoglycans: a novel strategy for high coverage and site-specific identification of glycosaminoglycan-linked peptides. Mol. Cell Proteom..

[36] Persson A, Nilsson J, Vorontsov E, Noborn F, Larson G (2019). Identification of a non-canonical chondroitin sulfate linkage region trisaccharide. Glycobiol..

[37] Pacheco B, Malmström A, Maccarana M (2009). Two dermatan sulfate epimerases form iduronic acid domains in dermatan sulfate. J. Biol. Chem..

[38] Tykesson E (2018). Dermatan sulfate epimerase 1 and dermatan 4-O-sulfotransferase 1 form complexes that generate long epimerized 4-O-sulfated blocks. J. Biol. Chem..

[39] Tykesson E (2016). Deciphering the mode of action of the processive polysaccharide modifying enzyme dermatan sulfate epimerase 1 by hydrogen–deuterium exchange mass spectrometry. Chem. Sci..

[40] Spencer JL, Bernanke JA, Buczek-Thomas JA, Nugent MA (2010). A computational approach for deciphering the organization of glycosaminoglycans. PLOS ONE.

[41] Zaia J (2016). Complete molecular weight profiling of low-molecular weight heparins using size exclusion chromatography-ion suppressor-high-resolution mass spectrometry. Anal. Chem..

[42] Gill VL, Aich U, Rao S, Pohl C, Zaia J (2013). Disaccharide analysis of glycosaminoglycans using hydrophilic interaction chromatography and mass spectrometry. Anal. Chem..

[43] Wu J (2019). Sequencing heparan sulfate using HILIC LC-NETD-MS/MS. Anal. Chem..

[44] Lawrence R (2008). Evolutionary Differences in Glycosaminoglycan Fine Structure Detected by Quantitative Glycan Reductive Isotope Labeling. J. Biol. Chem..

[45] Kailemia MJ, Li L, Ly M, Linhardt RJ, Amster IJ (2012). Complete mass spectral characterization of a synthetic ultralow-molecular-weight heparin using collision-induced dissociation. Anal. Chem..

[46] Zaia J, Li X-Q, Chan S-Y, Costello CE (2003). Tandem mass spectrometric strategies for determination of sulfation positions and uronic acid epimerization in chondroitin sulfate oligosaccharides. J. Am. Soc. Mass. Spectrom..

[47] Zamfir A, Seidler DG, Kresse H, Peter-Katalinić J (2003). Structural investigation of chondroitin/dermatan sulfate oligosaccharides from human skin fibroblast decorin. Glycobiol..

[48] Kudelka MR (2015). Cellular O-Glycome Reporter/Amplification to explore O-glycans of living cells. Nat. Methods.

[49] Zhao X (2013). Sequence analysis and domain motifs in the porcine skin decorin glycosaminoglycan chain. J. Biol. Chem..

[50] Fritz TA, Lugemwa FN, Sarkar AK, Esko JD (1994). Biosynthesis of heparan sulfate on β-D-xylosides depends on aglycone structure. J. Biol. Chem..

[51] Farndale RW, Sayers CA, Barrett AJ (1982). A Direct Spectrophotometric Microassay for Sulfated Glycosaminoglycans in Cartilage Cultures. Connect. Tissue Res..

[52] Vizcaíno JA (2016). 2016 update of the PRIDE database and its related tools. Nucleic Acids Res..

[53] Evers MR, Xia G, Kang H-G, Schachner M, Baenziger JU (2001). Molecular Cloning and Characterization of a Dermatan-specific N-Acetylgalactosamine 4-O-Sulfotransferase. J. Biol. Chem..

