# Supplementary material for: Glycosaminoglycan Domain Mapping of Cellular Chondroitin/Dermatan Sulfates
Source: Sci Rep. 2020 Feb 26;10:3506. doi: 10.1038/s41598-020-60526-0 (PMC7044218; doi:10.1038/s41598-020-60526-0)
Supplement: Supplementary file 1 — Supplementary information. [file 41598_2020_60526_MOESM1_ESM.pdf]

Supplementary information

Glycosaminoglycan Domain Mapping of Cellular Chondroitin/Dermatan Sulfates

Andrea Persson, Egor Vorontsov, Göran Larson, and Jonas Nilsson

Figures S1–S17, Tables S1–S3

Figures

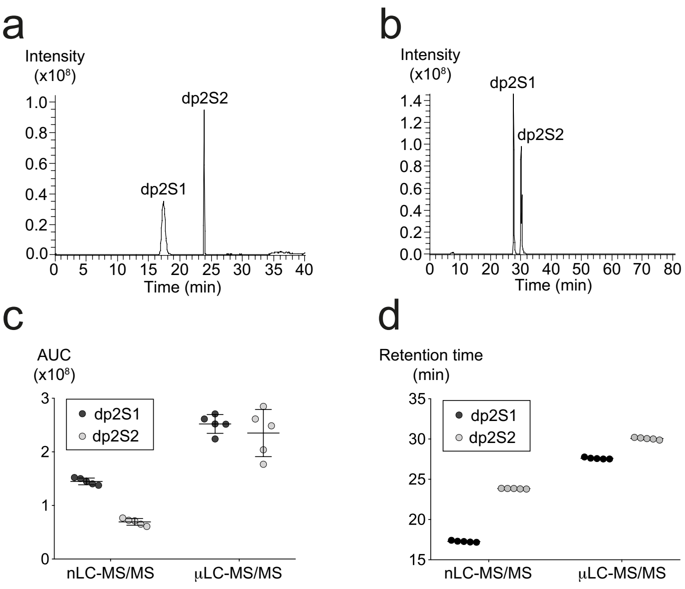

Figure S1. Comparison of nLC-MS/MS with μLC-MS/MS using disaccharide standards.

a,**b**, Base peak chromatograms of monosulfated (dp2S1) and disulfated (dp2S2) disaccharide standards at *m/z* 300–1,000 using nLC-MS/MS, the analytical setup in GAGDoMa, (**a**) and μLC-MS/MS (**b**). Disaccharide standards were run at 200 pg and 100 ng in nLC-MS/MS and μLC-MS/MS, respectively. **c**,**d**, Reproducibility with respect to intensity (**c**) and retention time (**d**). Chromatograms in **a** and **b** are representatives for each method. Data points are the means ±SD, where n=5 separate runs.

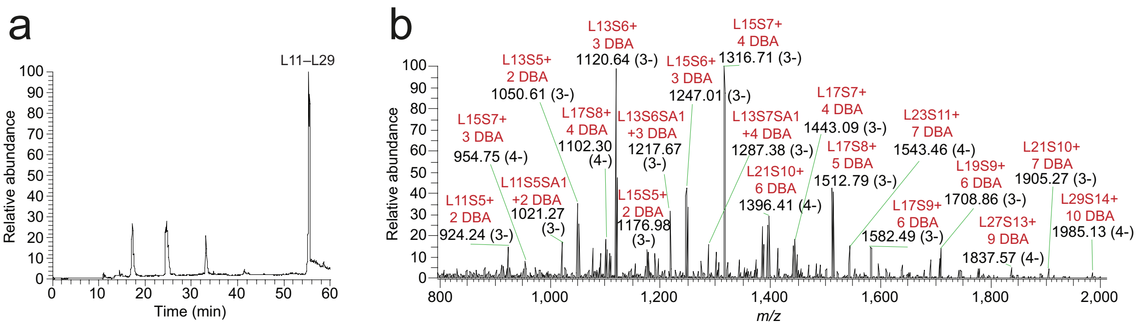

Figure S2. Identification of intact xyloside-primed CS/DS using GAGDoMa.

a, Total ion chromatogram of heparinase II and III-depolymerized xyloside-primed GAGs from HCC70 cells. **b**, MS[1] spectra at 55.22–55.58 min (12 spectra) of heparinase II and III-depolymerized xyloside-primed GAGs from HCC70 cells displaying intact CS/DS structures. DBA, dibutylamine. SA, sialic acid.

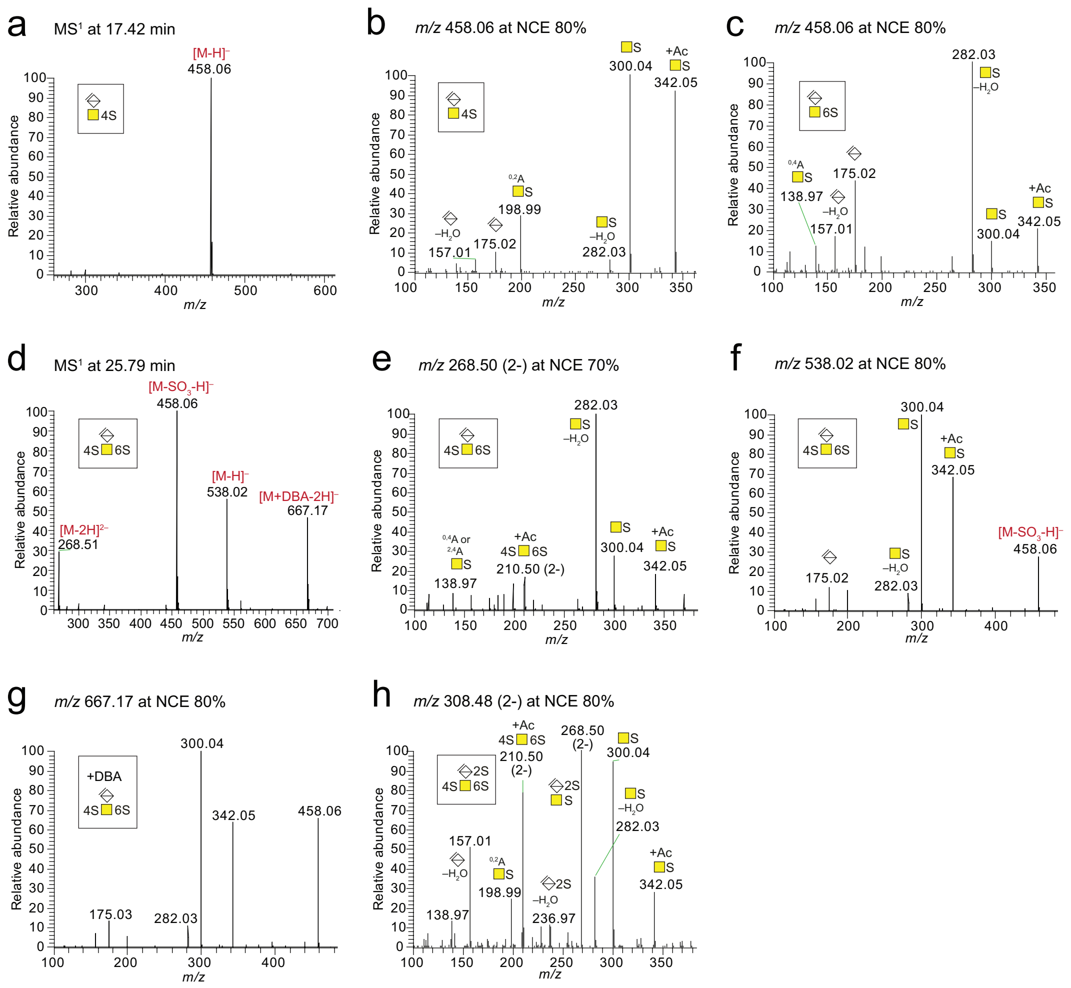

Figure S3. Internal disaccharide variants.

a, MS[1] spectra at 17.42 min of chondroitinase B-depolymerized xyloside-primed CS/DS displaying the $[M\text{-}H]^-$ precursor ion at *m/z* 458.06 corresponding to the ΔHexA-GalNAc4S disaccharide. **b**, MS[2] spectra at *m/z* 458.06 at normalized collision energy (NCE) 80% of ΔHexA-GalNAc4S from CCD-1095Sk cells after chondroitinase B depolymerization. **c**, MS[2] spectra at *m/z* 458.06 at NCE 80% of ΔHexA-GalNAc6S from HCC70 cells after chondroitinase AC depolymerization. **d**, MS[1] spectra at 25.79 min of the ΔHexA-GalNAc4S6S disaccharide standard displaying the four different precursor ions. **e**–**g**, MS[2] spectra of the ΔHexA-GalNAc4S6S disaccharide standard; the $[M\text{-}2H]^{2-}$ precursor ion at *m/z* 268.51 at NCE 70% (**e**), the $[M\text{-}H]^-$ precursor ion at *m/z* 538.02 at NCE 80% (**f**), and the $[M\text{+}DBA\text{-}2H]^-$ precursor ion at *m/z* 667.17 at NCE 80% (**g**). **h**, MS[2] spectrum of the $[M\text{-}2H]^{2-}$ precursor ion at *m/z* 308.48 at NCE 80% corresponding to ΔHexA2S-GalNAc4S6S. DBA, dibutylamine.

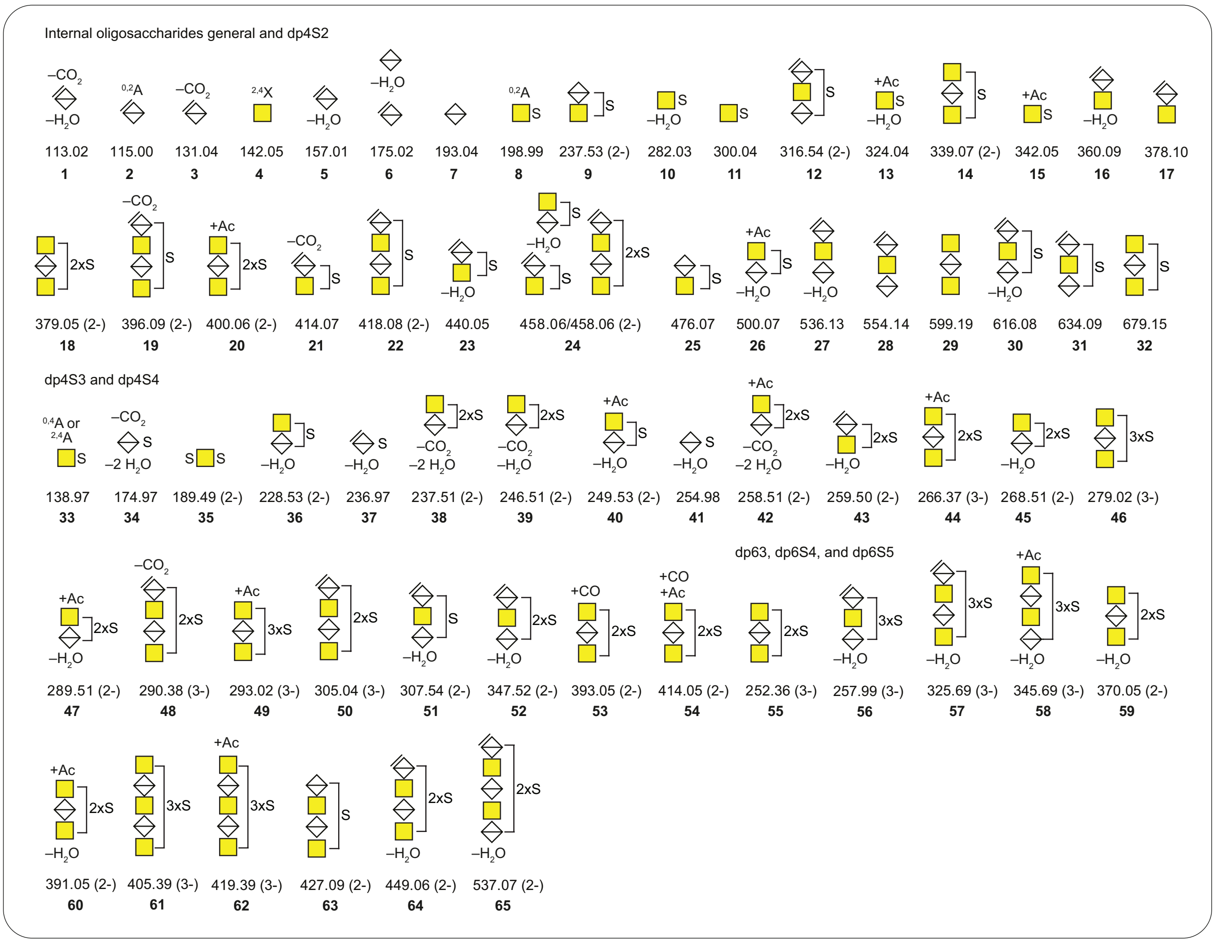

Figure S4. Annotated fragment ions of internal oligosaccharides.

Proposed annotation and numbering of ions generated from HCD fragmentation of internal oligosaccharide structures.

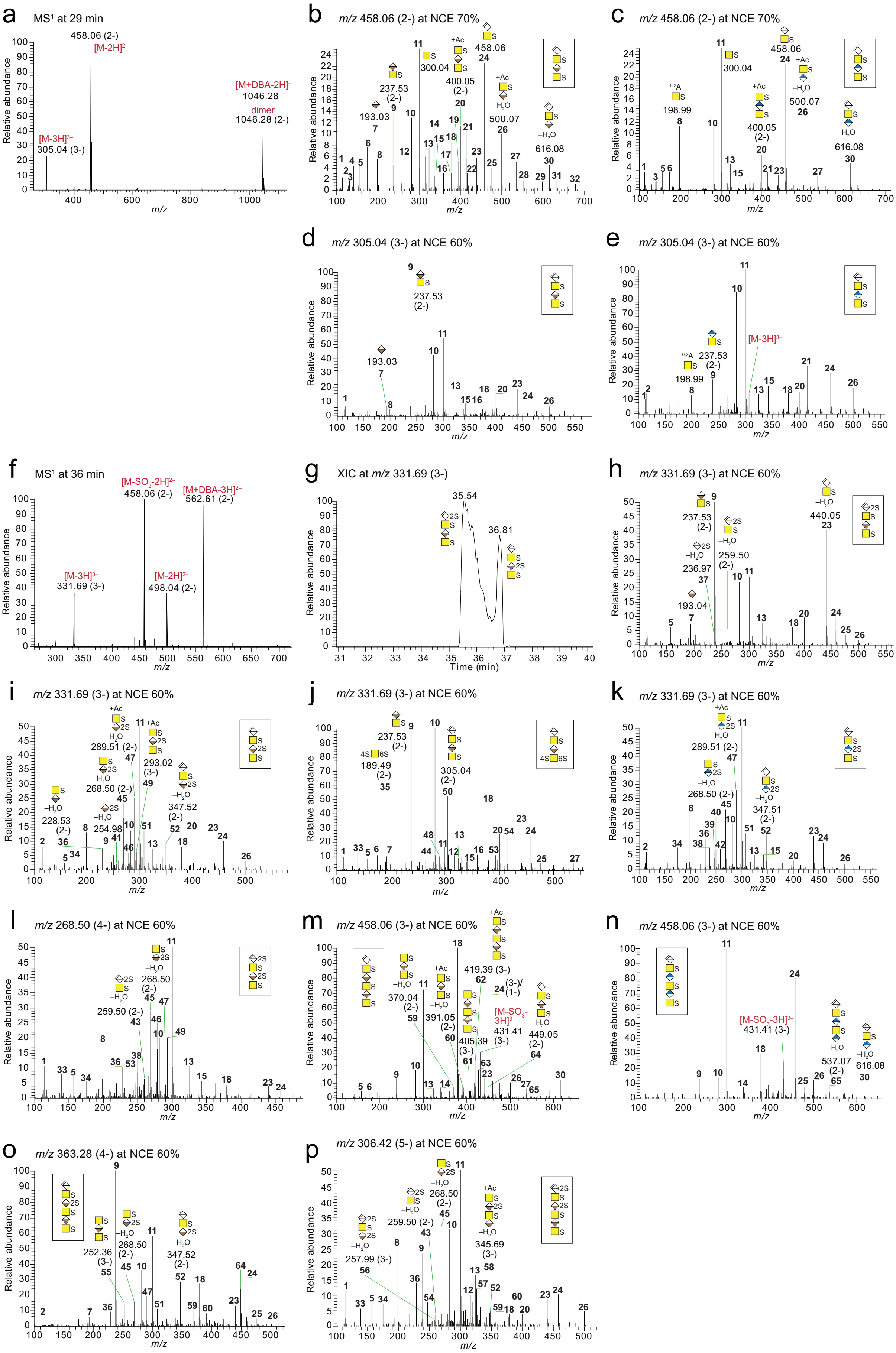
a MS¹ at 29 min
458.06 (2-)
[M-2H]²⁻
[M+DBA-2H]⁻
1046.28
dimer
1046.28 (2-)
[M-3H]³⁻
305.04 (3-)
b m/z 458.06 (2-) at NCE 70%
c m/z 458.06 (2-) at NCE 70%
d m/z 305.04 (3-) at NCE 60%
e m/z 305.04 (3-) at NCE 60%
f MS¹ at 36 min
[M-SO3-2H]²⁻
458.06 (2-)
[M+DBA-3H]²⁻
562.61 (2-)
[M-3H]³⁻
331.69 (3-)
[M-2H]²⁻
498.04 (2-)
g XIC at m/z 331.69 (3-)
35.54
36.81
Time (min)
h m/z 331.69 (3-) at NCE 60%
i m/z 331.69 (3-) at NCE 60%
j m/z 331.69 (3-) at NCE 60%
k m/z 331.69 (3-) at NCE 60%
l m/z 268.50 (4-) at NCE 60%
m m/z 458.06 (3-) at NCE 60%
n m/z 458.06 (3-) at NCE 60%
[M-SO3-3H]³⁻
431.41 (3-)
o m/z 363.28 (4-) at NCE 60%
p m/z 306.42 (5-) at NCE 60%
Relative abundance
m/z

Figure S5. Internal tetra- and hexasaccharide variants.

a, MS^1 spectrum at 29 min of chondroitinase AC-depolymerized xyloside-primed CS/DS from CCD-1095Sk cells displaying the dp4S2 precursor ions. **b**–**e**, MS^2 spectra of two dp4S2 isomers with respect to IdoA (**b**,**d**) and GlcA (**c**,**e**) displayed as the $[M\text{-}2H]^{2-}$ precursor ion *m/z* 458.06 at NCE 70% (**b**,**c**) and the $[M\text{-}3H]^{3-}$ precursor ion at *m/z* 305.04 at NCE 60% (**d** and **e**). **b**,**d** and **c**,**e** are from chondroitinase AC and B depolymerizations, respectively. **f**, MS^1 spectrum at 36 min of chondroitinase AC-depolymerized xyloside-primed CS/DS from CCD-1095Sk cells displaying the dp4S3 precursor ions. **g**, Extracted ion chromatogram (XIC) at *m/z* 331.69 displaying two distinct peaks corresponding to the isomers shown in **h** and **i**. **h**–**k**, MS^2 spectra of four dp4S3 isomers, with respect to sulfate group position and IdoA and GlcA, displayed as $[M\text{-}3H]^{3-}$ precursor ions at *m/z* 331.69 at NCE 60%. **h**–**j** and **k** are from chondroitinase AC and B depolymerizations, respectively. **l**, MS^2 spectrum of the $[M\text{-}4H]^{4-}$ precursor ion at *m/z* 268.50 at NCE 60% corresponding to dp4S4 where each monosaccharide carries a sulfate group. **m**,**n**, MS^2 spectra of two dp6S3 isomers with respect to IdoA (**m**) and GlcA (**n**) displayed as the $[M\text{-}3H]^{3-}$ precursor ion *m/z* 458.06 at NCE 60%. **m** and **n** are from chondroitinase AC and B depolymerizations, respectively. **o**, MS^2 spectrum of the $[M\text{-}4H]^{4-}$ precursor ion at *m/z* 363.28 at NCE 60% corresponding to dp6S4 where each GalNAc residue and the HexA residue towards the non-reducing end (NRE) carry a sulfate group. **p**, MS^2 spectrum of the $[M\text{-}5H]^{5-}$ precursor ion at *m/z* 306.42 at NCE 60% corresponding to dp6S5 where each monosaccharide except the IdoA residue towards the reducing end carries a sulfate group. Critical fragment ions are indicated by glycan symbols and the remaining ions by the numerals given in Figure S4.

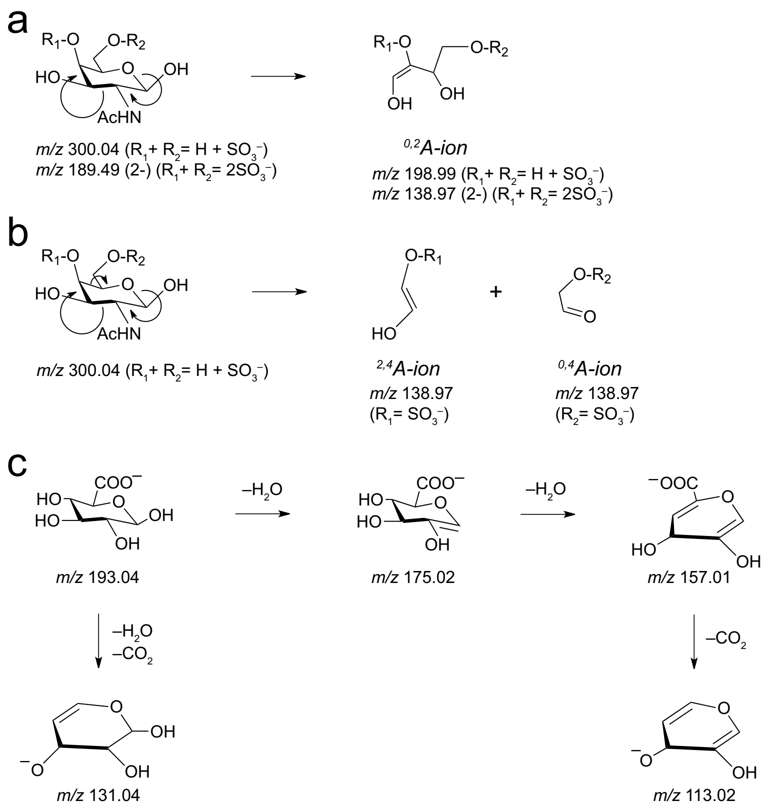

Figure S6. Fragmentation pathways for GalNAc and HexA residues.

a,**b**, Proposed fragmentation reactions for *0,2A* cross-ring cleavage (**a**); and *2,4A* and *0,4A* cross-ring cleavages (**b**) of the sulfated GalNAc residue. **c**, proposed structures of fragment ions generated from the HexA residue.

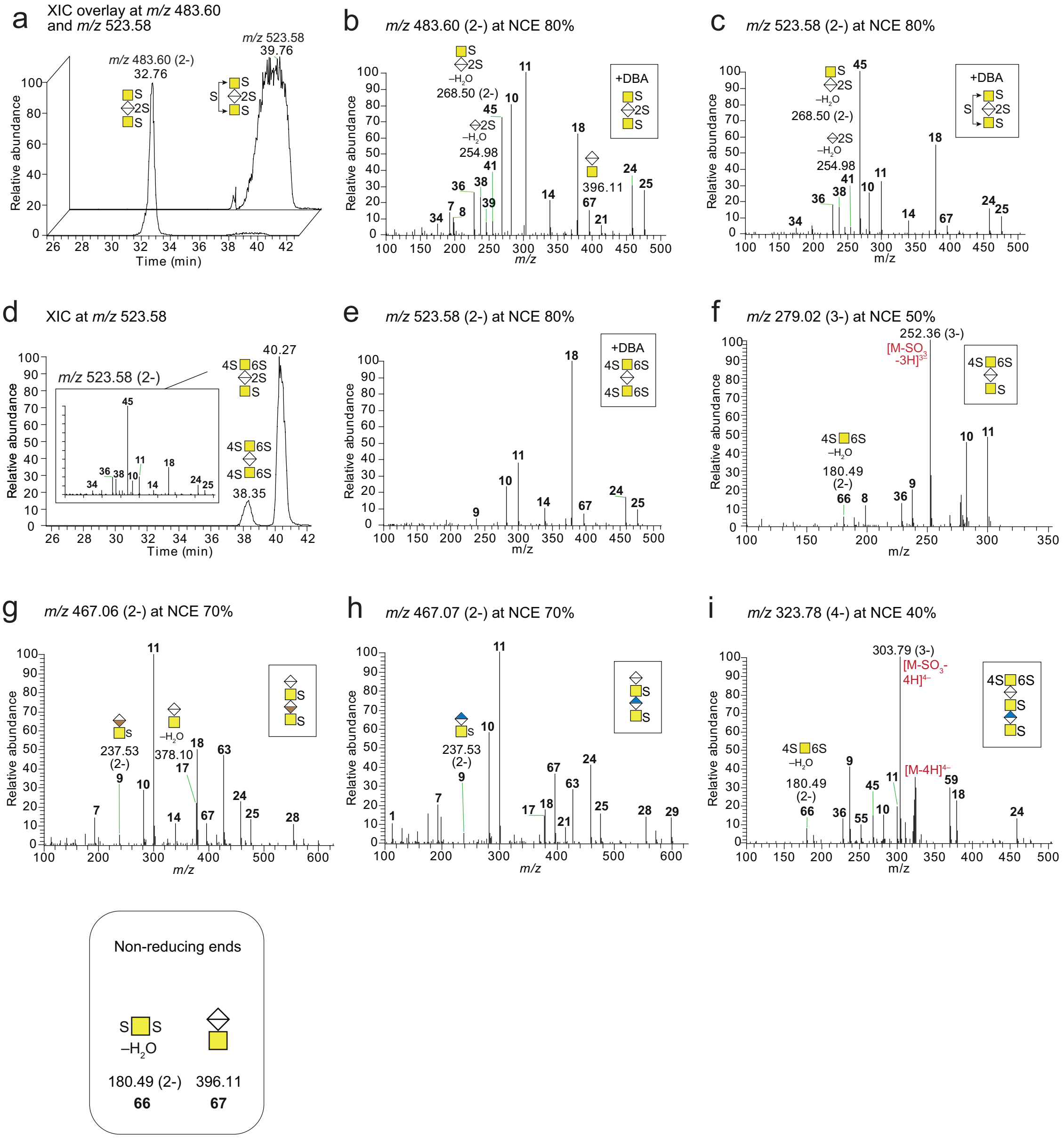

Figure S7. Terminal non-reducing end variants.

Chromatograms and spectra of the different variants, including an annotation chart for fragment ions specific for the structures. **a**, XIC overlay at *m/z* 483.60 and *m/z* 523.58 of chondroitinase AC-depolymerized xyloside-primed CS/DS from CCD-1095Sk cells displaying dp3S3 and dp3S4 NRE variants shown in **b** and **c**. **b**, MS^2 spectrum of the $[M-2H]^{2-}$ precursor ion at *m/z* 483.60 at NCE 80% corresponding to dp3S3 where each monosaccharide carries one sulfate group. **c**, MS^2 spectrum of the $[M-2H]^{2-}$ precursor ion at *m/z* 523.58 at NCE 80% corresponding to dp3S4 where each monosaccharide carries one sulfate group and one of the GalNAc residues carries an additional sulfate group. **d**, XIC at *m/z* 523.58 of chondroitinase AC-depolymerized xyloside-primed CS/DS from HCC70 cells displaying dp3S4 NRE variants shown in the inset and **e**. **e**, MS^2 spectrum of the [M-2H]$^{2-}$ precursor ion at *m/z* 523.58 at NCE 80% corresponding to dp3S4 where each GalNAc residue carries two sulfate groups. **f**, MS^2 spectrum of the $[M-3H]^{3-}$ precursor ion at *m/z* 279.02 at NCE 50% corresponding to dp3S3 where the NRE GalNAc residue carries two sulfate groups and the reducing

end GalNAc residue carries one sulfate group. **g**,**h**, MS^2 spectra of the $[M\text{-}2H]^{2-}$ precursor ions at *m/z* 467.06 at NCE 70% corresponding to NRE dp4S2 after chondroitinase AC (**g**) and B (**h**) depolymerizations where each GalNAc residue carries one sulfate group. $^{0,2}X$ cleavages of NRE precursor ions do not appear to any great extent; **15** was the only observed $^{0,2}X$-ion for the NRE dp4S2 isomers and its relative abundance was 1.5% and 0.5% in **h** and **g,** respectively. The relative abundance of the internal dp4S2 isomers were 24.4% and 29.7% (total relative abundance of $^{0,2}X$-ions **13**, **15**, **20**, and **26**) in Figures S2**b** and S2**c**, respectively. This further supports that the fragmentation occurs towards the NRE, generating $^{0,2}X$-ions, rather than towards the reducing end, generating $^{2,4}A$-ions, which has been suggested for Na^+/H^+ exchange CID fragmentation[1]. **i**, MS^2 spectrum of the $[M\text{-}4H]^{4-}$ precursor ion at *m/z* 323.78 at NCE 40% corresponding to dp5S4 where the NRE GalNAc residue carries two sulfate groups and the other two carry one sulfate group each. Critical fragment ions are indicated by glycan symbols and the remaining ions by the numerals given in Figure S4 and the present figure.

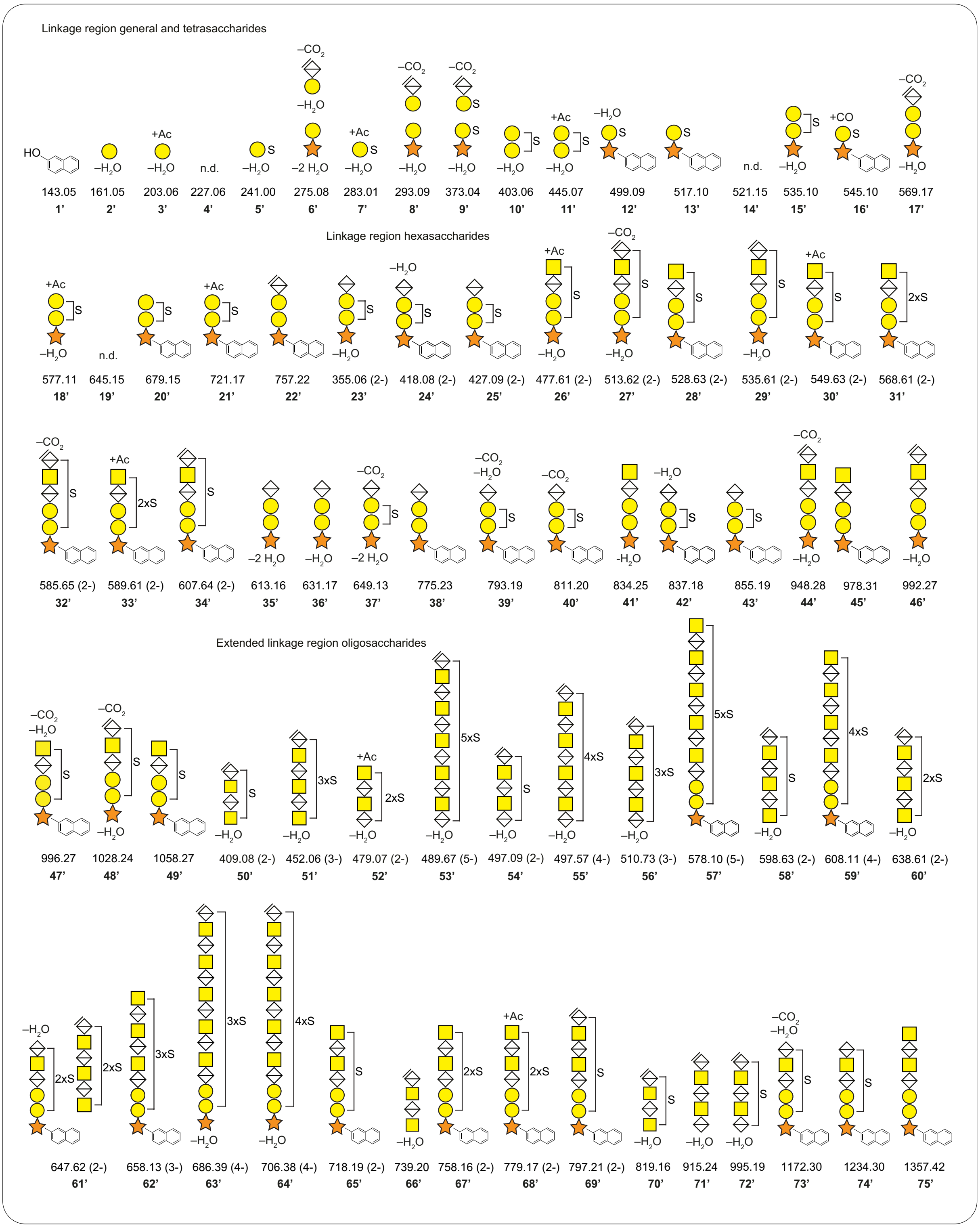

Figure S8. Annotated fragment ions of linkage regions.

Proposed annotation and numbering of ions generated from fragmentation of linkage region structures. n.d., not determined.

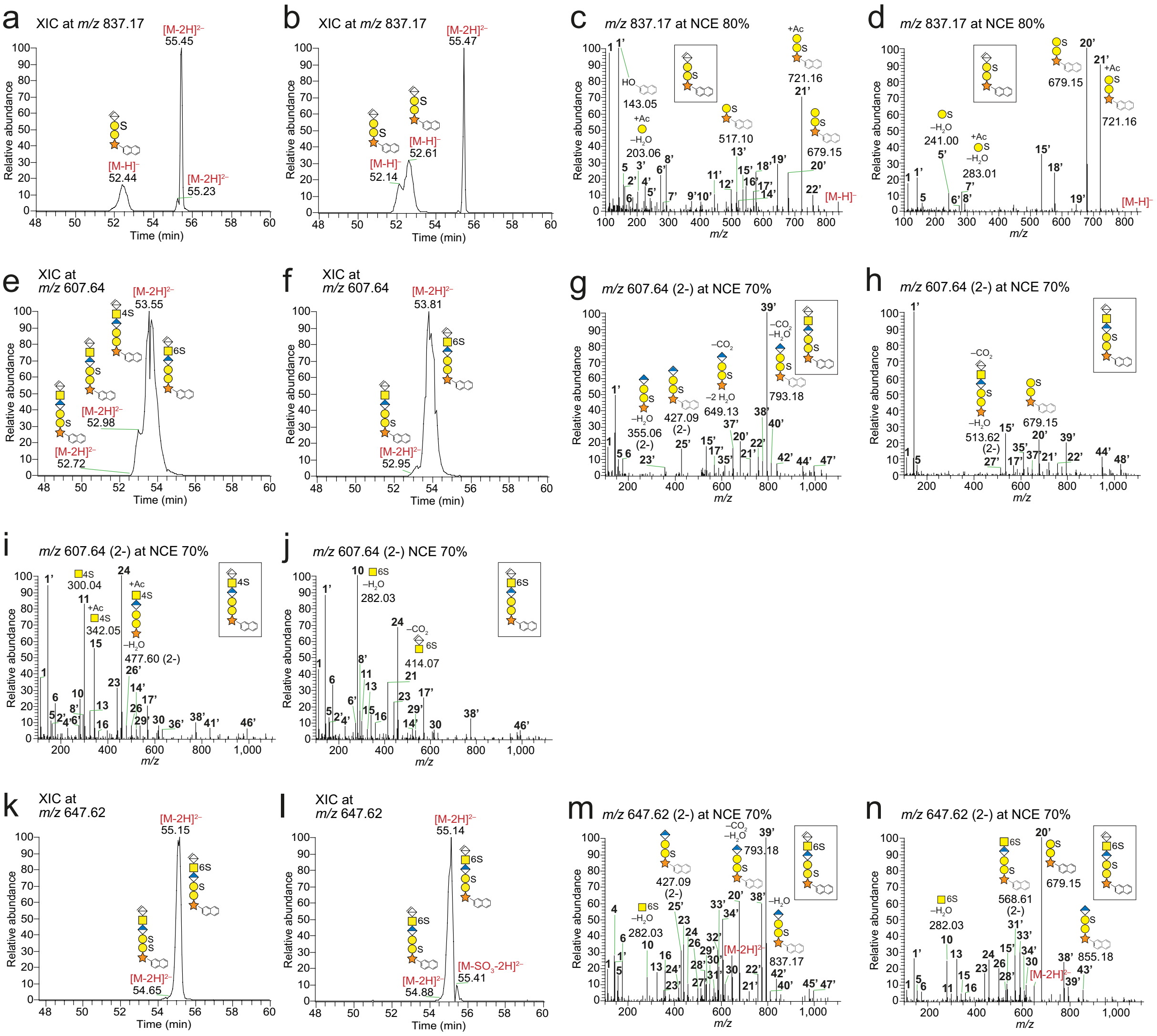

Figure S9. Linkage region tetra- and hexasaccharide variants.

a,**b**, XIC at *m/z* 837.17 of chondroitinase AC-depolymerized xyloside-primed CS/DS from CCD-1095Sk cells (**a**) and HCC70 cells (**b**) displaying ΔL4S1 isomers shown in **c** and **d**. **c**,**d**, MS^2 spectra of the [M-H]$^–$ precursor ion at *m/z* 837.17 at NCE 80% corresponding to the two different ΔL4S1 isomers. **e**,**f**, XIC at *m/z* 607.64 of chondroitinase AC-depolymerized xyloside-primed CS/DS from CCD-1095Sk cells (**e**) and HCC70 cells (**f**) displaying ΔL6S1 isomers shown in **g**–**j**. **g**–**j**, MS^2 spectra of the [M-2H]$^{2–}$ precursor ion at *m/z* 607.64 at NCE 70% corresponding to four different ΔL6S1 isomers. Distinction of the isomers in **i** and **j** was performed based on dominating fragment ions at *m/z* 300.04 and *m/z* 282.03 assigning the sulfate group to the GalNAc residue at position 4 (**i**) and 6 (**j**), respectively. **k**,**l**, XIC at *m/z* 647.62 of chondroitinase AC-depolymerized xyloside-primed CS/DS from CCD-1095Sk cells (**k**) and HCC70 cells (**l**) displaying ΔL6S2 isomers shown in **m** and **n**. **m**,**n**, MS^2 spectra of the [M-2H]$^{2–}$ precursor ion at *m/z* 647.62 at NCE 70% corresponding to two different ΔL6S2 isomers. Critical fragment ions are indicated by glycan symbols and the remaining ions by the numerals given in Figures S4 and S8.

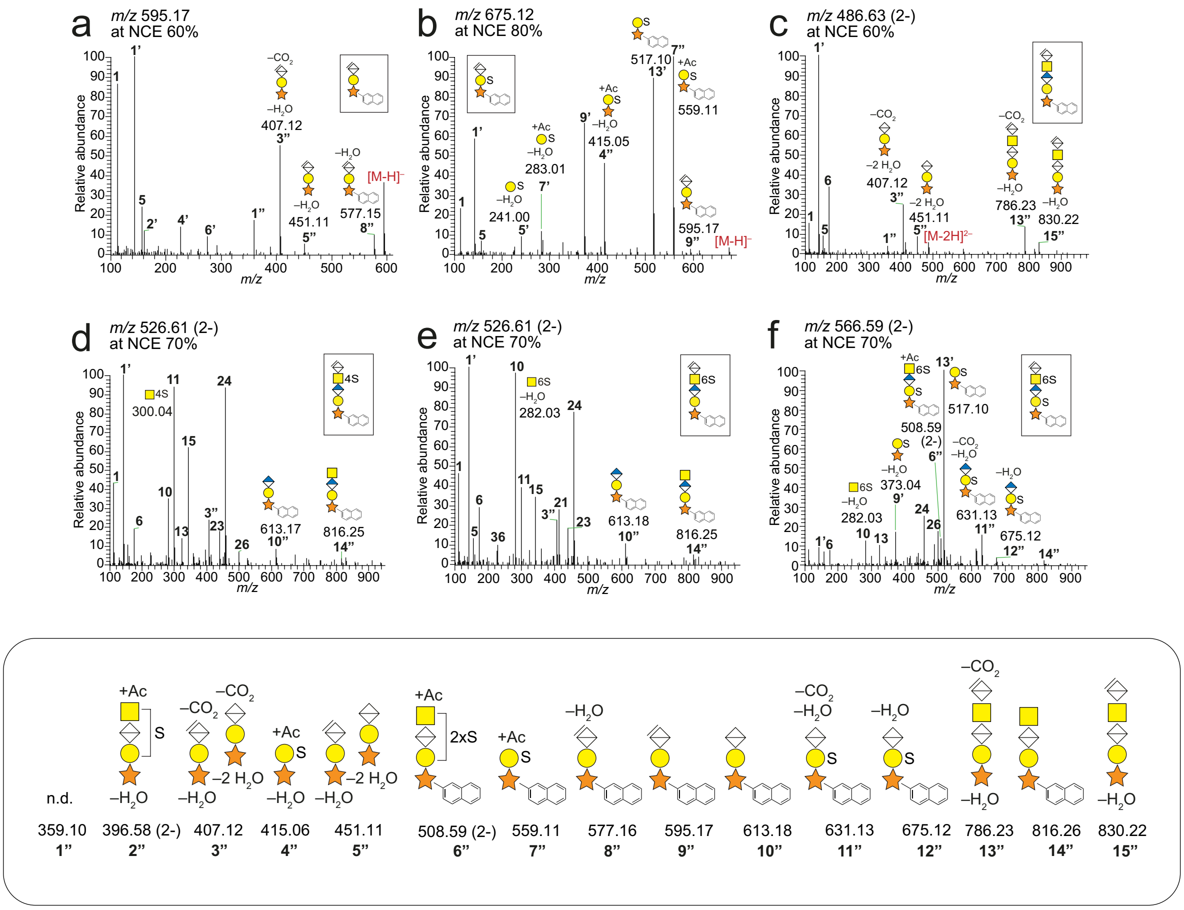

Figure S10. Linkage region tri- and pentasaccharide variants.

Spectra of variants containing a non-canonical linkage region trisaccharide core structure, including an annotation chart for fragment ions specific for the structures. **a**, MS^2 spectrum of the [M-H]$^-$ precursor ion at *m/z* 595.17 at NCE 60% corresponding to ΔL3S0. **b**, MS^2 spectrum of the [M-H]$^-$ precursor ion at *m/z* 675.12 at NCE 80% corresponding to ΔL3S1. **c**, MS^2 spectrum of the [M-2H]$^{2-}$ precursor ion at *m/z* 486.63 at NCE 60% corresponding to ΔL5S0. **d**,**e**, MS^2 spectra of the [M-2H]$^{2-}$ precursor ions at *m/z* 526.61 at NCE 70% corresponding to ΔL5S1. Distinction of the isomers was performed based on dominating fragment ions at *m/z* 300.04 and *m/z* 282.03 assigning the sulfate group to the GalNAc residue at position 4 (**d**) and 6 (**e**), respectively. **f**, MS^2 spectrum of the [M-2H]$^{2-}$ precursor ion at *m/z* 566.59 at NCE 70% corresponding to ΔL5S2. Critical fragment ions are indicated by glycan symbols and the remaining ions by the numerals given in Figures S4, S8, and in the annotation chart in the figure. n.d., not determined.

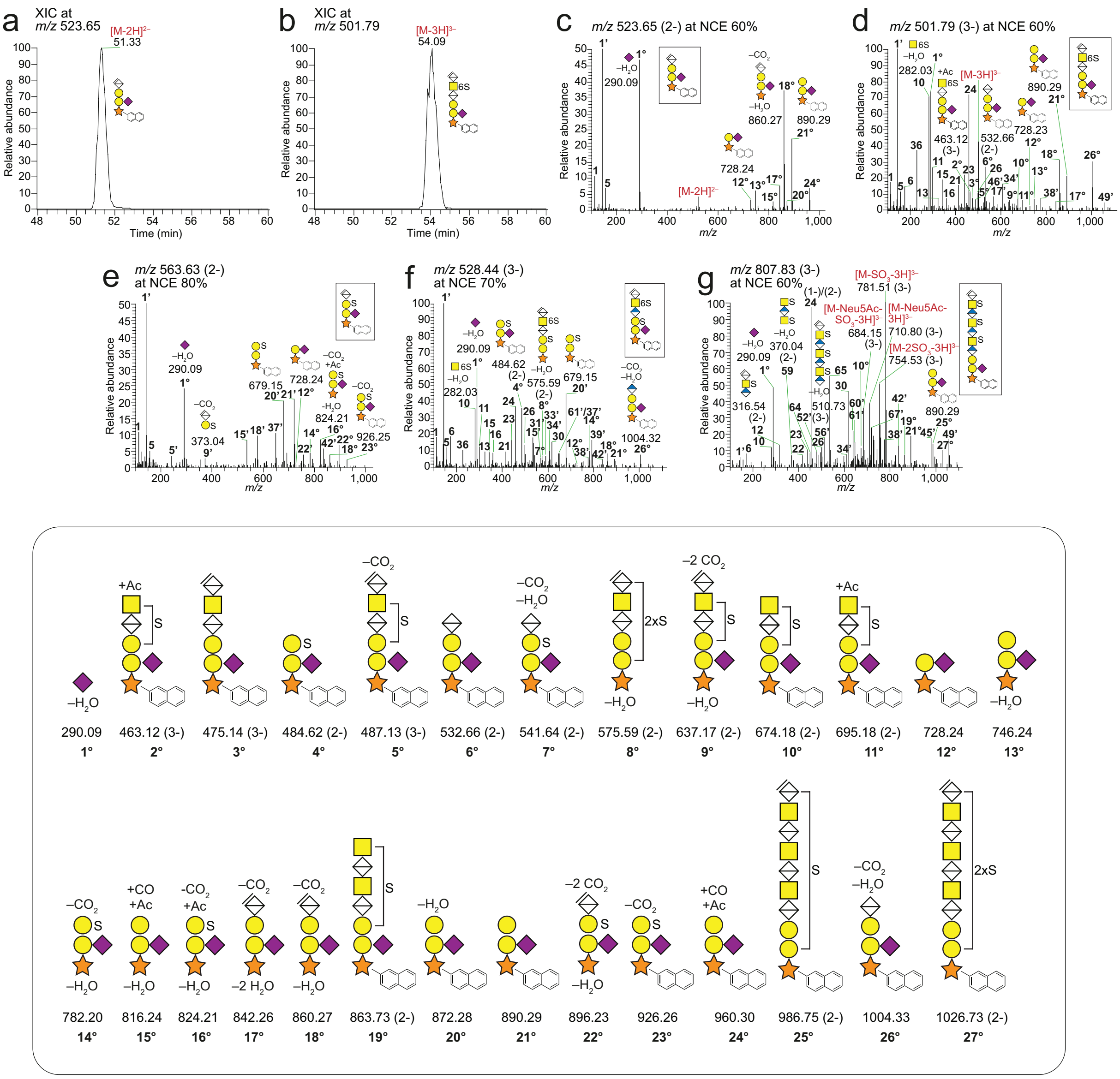

Figure S11. Sialylated linkage region variants.

Chromatograms and spectra of the different sialylated variants, including an annotation chart for fragment ions specific for the structures. **a**,**b**, XIC at *m/z* 523.65 of chondroitinase AC-depolymerized (**a**) and *m/z* 501.79 of chondroitinase ABC-depolymerized (**b**) xyloside-primed CS/DS from HCC70 cells displaying the variants fragmented in **c** and **d**. **c**, MS^2 spectrum of the [M-2H]$^{2-}$ precursor ion at *m/z* 523.65 at NCE 60% corresponding to ΔL4SA1. **d**, MS^2 spectrum of the [M-3H]$^{3-}$ precursor ion at *m/z* 501.79 at NCE 60% corresponding to ΔL6S1SA1. **e**, MS^2 spectrum of the [M-2H]$^{2-}$ precursor ion at *m/z* 563.63 at NCE 80% corresponding to ΔL4S1SA1. **f**, MS^2 spectrum of the [M-3H]$^{3-}$ precursor ion at *m/z* 528.44 at NCE 70% corresponding to ΔL6S2SA1. **g**, MS^2 spectrum of the [M-3H]$^{3-}$ precursor ion at *m/z* 807.83 at NCE 60% corresponding to ΔL10S3SA1. The suggested loss of CO_2 from the Neu5Ac residue has previously been described for negative mode MS/MS of N-glycans[2]. Critical fragment ions are indicated by glycan symbols and the remaining ions by the numerals given in Figures S4, S8, and in the annotation chart in the figure.

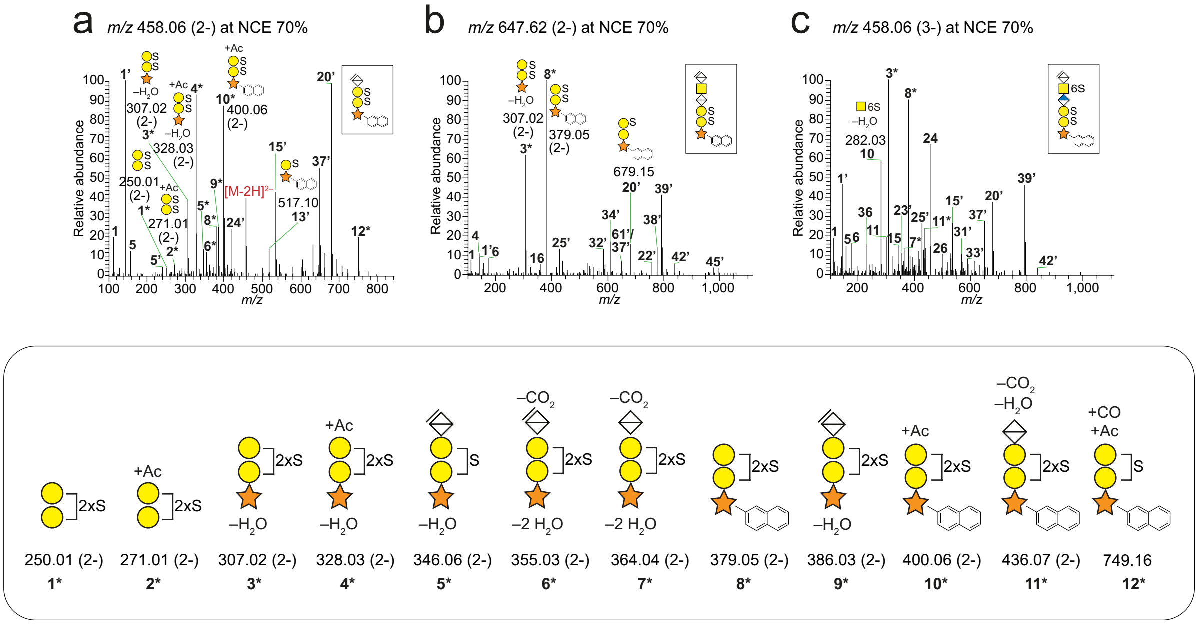

Figure S12. Linkage region variants containing two sulfate groups on the Gal residues. Spectra of the different variants, including an annotation chart for fragment ions specific for the structures. **a**, MS^2 spectrum of the $[M-2H]^{2-}$ precursor ion at *m/z* 458.06 at NCE 70% corresponding to ΔL4S2. **b**, MS^2 spectrum of the $[M-2H]^{2-}$ precursor ion at *m/z* 647.62 at NCE 70% corresponding to ΔL6S2. **c**, MS^2 spectrum of the $[M-3H]^{3-}$ precursor ion at *m/z* 458.06 at NCE 60% corresponding to ΔL6S3. We cannot with certainty exclude that both sulfate groups are positioned at one of the Gal residues; however, there are no fragment ions present supporting such a structure. Critical fragment ions are indicated by glycan symbols and the remaining ions by the numerals given in Figures S4, S8, and in the annotation chart in the figure.

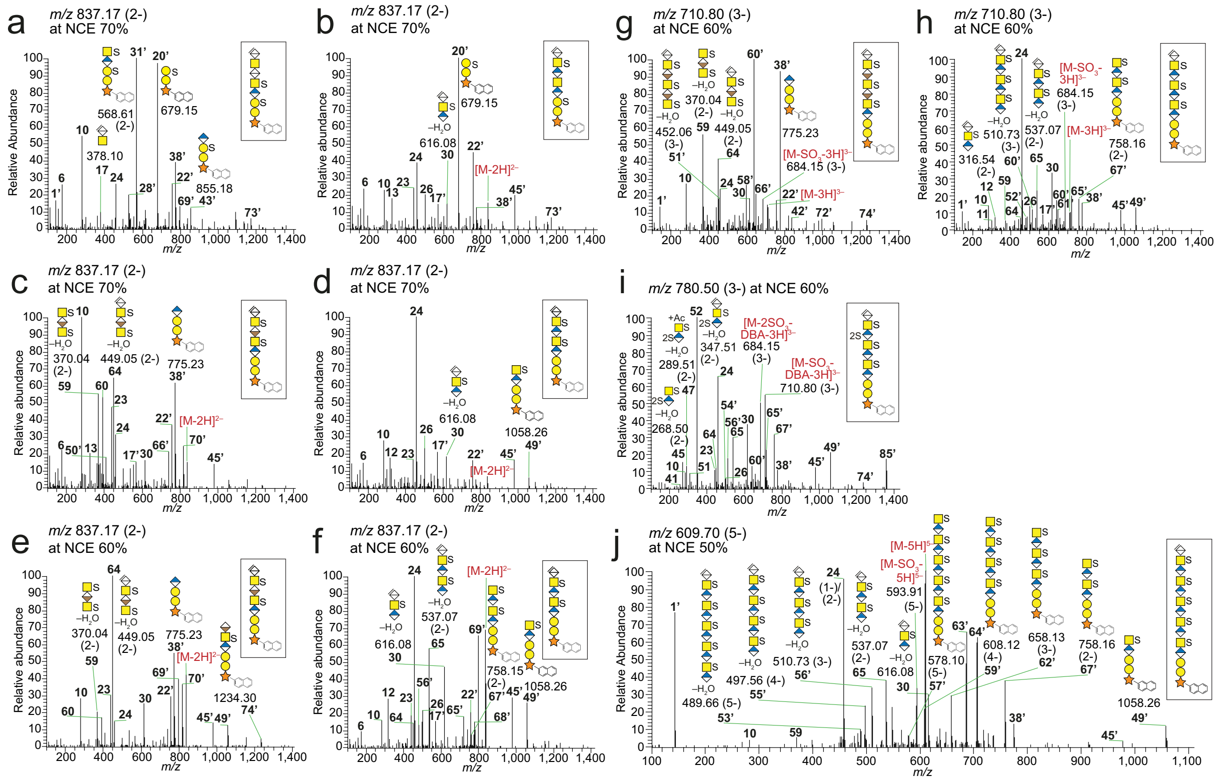

Figure S13. Extended linkage region variants.

a–**f**, MS^2 spectra of the $[M\text{-}2H]^{2-}$ precursor ion at *m/z* 837.17 corresponding to different ΔL8S2 isomers. **c** and **e** correspond to the same structure, and **d** and **f** correspond to the same structure, only fragmented at different energies; NCE 70% (**c** and **e**), and NCE 60% (**d** and **f**). These examples were included to display that GlcA containing structures appears more susceptible to fragmentation, and therefore require lower NCEs than IdoA containing structures. **g**,**h**, MS^2 spectra of the $[M\text{-}3H]^{3-}$ precursor ion at *m/z* 710.80 at NCE 60% corresponding to two ΔL10S3 isomers. **i**, MS^2 spectra of the $[M\text{+}DBA\text{-}4H]^{3-}$ precursor ion at *m/z* 780.50 at NCE 60% corresponding to ΔL10S4 where one sulfate group is pinpointed to the first GlcA from the NRE and the remaining to each GalNAc residue. **j**, MS^2 spectra of the $[M\text{-}5H]^{5-}$ precursor ion at *m/z* 609.70 at NCE 50% corresponding to ΔL14S5 where one sulfate group is pinpointed to each GalNAc residue. Critical fragment ions are indicated by glycan symbols and the remaining ions by the numerals given in Figures S4 and S8.

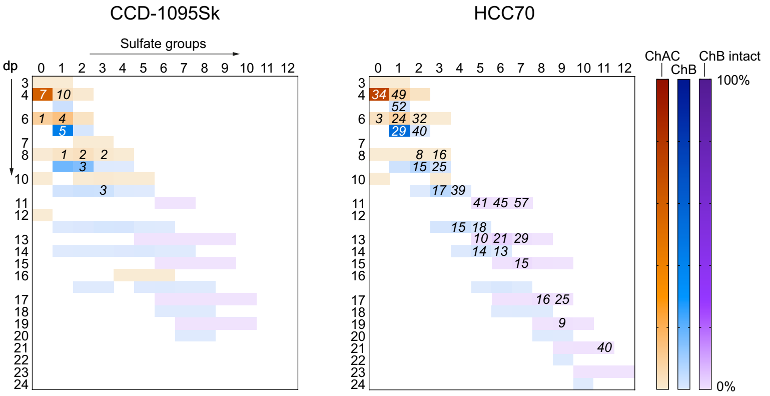

Figure S14. Amounts of sialylated linkage region variants. Linkage region heat map summary including the relative amount of each variant carrying a Neu5Ac residue, calculated using the area under the curve of the precursor ion peaks. Colors and abbreviations are the same as in Figure 5 in the main text.

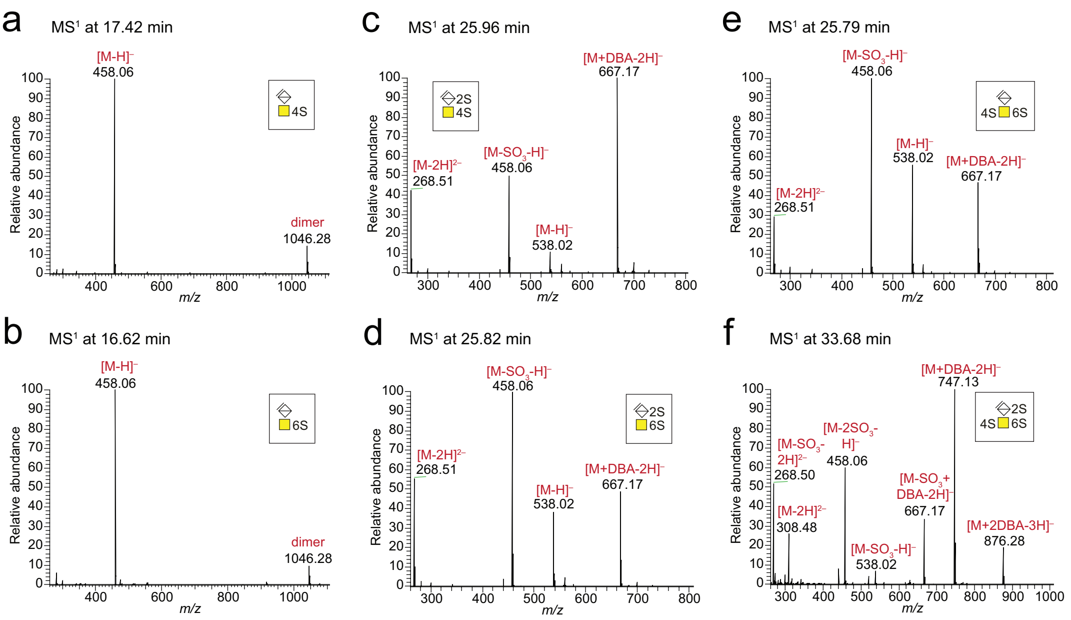

Figure S15. MS^1 of disaccharides. MS^1 spectra of the ΔHexA-GalNAc4S (**a**), ΔHexA-GalNAc6S (**b**), ΔHexA2S-GalNAc4S (**c**), ΔHexA2S-GalNAc6S (**d**), ΔHexA-GalNAc4S6S (**e**), and ΔHexA2S-GalNAc4S6S (**f**) disaccharides. The relative in-source sulfate loss is 0% for the two monosulfated disaccharides (**a, b**), 24% for ΔHexA2S-GalNAc4S (**c**), 41% for ΔHexA2S-GalNAc6S (**d**), 43% for ΔHexA-GalNAc4S6S (**e**), and 51% for ΔHexA2S-GalNAc4S6S (**f**).

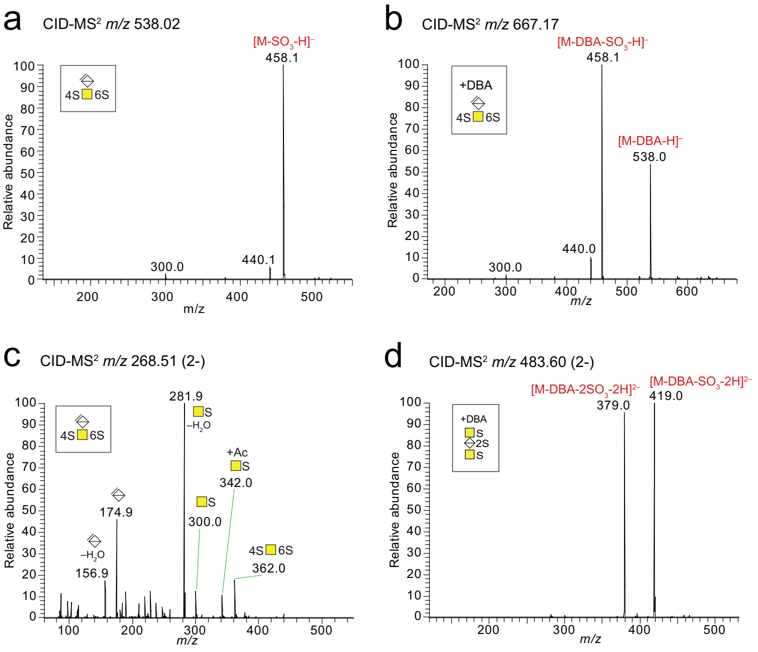

Figure S16. Oligosaccharide fragmentation using collision-induced dissociation (CID). a–c, MS^2 spectra of the ΔHexA-GalNAc4S6S disaccharide standard, the [M-H]$^-$ precursor ion at *m/z* 538.02 (**a**), the [M+DBA-2H]$^-$ precursor ion at *m/z* 667.17 (**b**), and the [M-2H]$^{2-}$ precursor ion at *m/z* 268.51 (**c**). **d**, MS^2 spectrum of the [M+DBA-3H]$^{2-}$ precursor ion of the NRE dp3S3 oligosaccharide at *m/z* 483.60. In addition to the generation of less informative fragment ions compared to HCD, the mass accuracy of CID is poor. The CID-MS^2 spectra in **a**–**d** were acquired at NCE 60%.

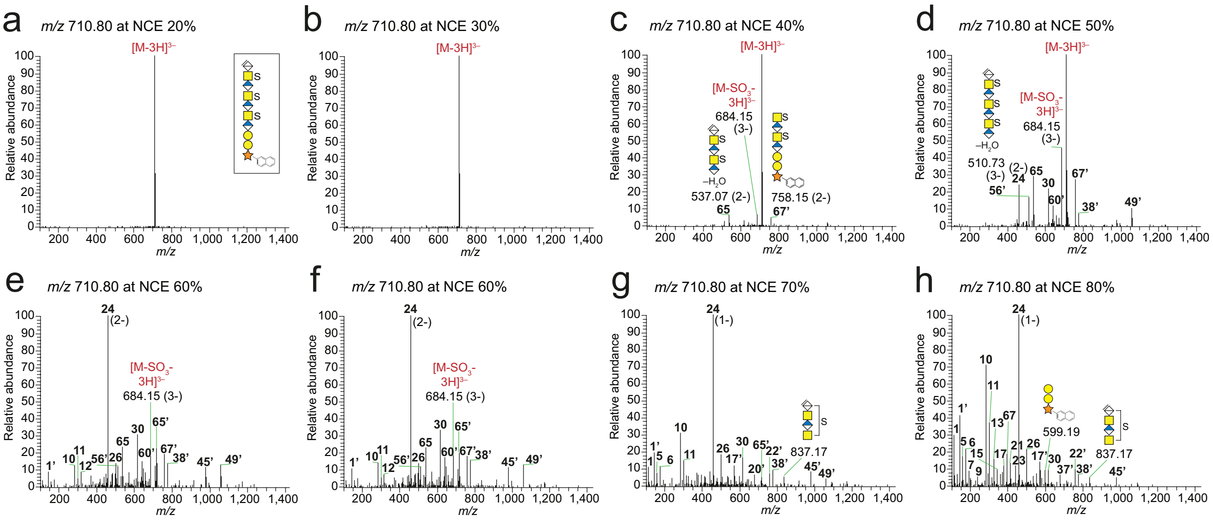

Figure S17. Fragmentation patterns of ΔL10S3 at different NCE levels. MS^2 spectra of the [M-3H]$^{3-}$precursor ion at *m/z* 710.80 at NCE of 20% (**a**), 30% (**b**), 40% (**c**), 50% (**d**), 60% (**e**), 60% (**f**), 70% (**g**), and 80% (**h**). The spectra in **e** and **f** are from two separate runs demonstrating the reproducibility of the fragmentation between runs.

Tables

Table S1. Compositional profiling of intact xyloside-primed CS/DS.

Glycan composition	Experimental mass[1]	Theoretical mass	*z*	Acc. (ppm)	Reference file
L11S4 + 1 DBA	854.1998	854.2028	3	-3.6	HCC_Hep_I
L11S5 + 2 DBA	923.9026	923.9057	3	-3.4	HCC_Hep_I
L11S6 + 3 DBA	993.6050	993.6086	3	-3.6	HCC_Hep_I
L11S7 + 4 DBA	1063.3103	1063.3114	3	-1.1	HCC_Hep_I
L11S5SA1 + 2 DBA	1020.9343	1020.9375	3	-3.1	HCC_Hep_I
L11S6SA1 + 3 DBA	1090.6365	1090.6404	3	-3.5	HCC_Hep_II
L11S7SA1 + 4 DBA	1160.3385	1160.3432	3	-4.1	HCC_Hep_II
L12S4 + 1 DBA	912.8765	912.8802	3	-4.1	HCC_Hep_I
L12S5 + 2 DBA	982.5790	982.5831	3	-4.1	HCC_Hep_I
L13S5 + 2 DBA	1050.2723	1050.2762	3	-3.7	HCC_Hep_I
L13S6 + 3 DBA	1119.9754	1119.9791	3	-3.3	HCC_Hep_I
L13S7 + 4 DBA	1189.6773	1189.6819	3	-3.9	HCC_Hep_I
L13S8 + 5 DBA	1259.3807	1259.3848	3	-3.3	HCC_Hep_I
L13S5SA1 + 3 DBA	1147.3033	1147.3080	3	-4.1	HCC_Hep_I
L13S6SA1 + 3 DBA	1217.0078	1217.0109	3	-2.5	HCC_Hep_I
L13S7SA1 + 4 DBA	1286.7087	1286.7137	3	-3.9	HCC_Hep_I
L13S8SA1 + 5 DBA	1356.4104	1356.4166	3	-4.6	HCC_Hep_I
L14S4 + 1 DBA	1039.2478	1039.2507	3	-2.8	HCC_Hep_I
L14S5 + 2 DBA	1108.9485	1108.9536	3	-4.6	HCC_Hep_I
L15S5 + 2 DBA	1176.6419	1176.6467	3	-4.1	HCC_Hep_I
L15S6 + 3 DBA;<br>L15S6 + 2 DBA	1246.3445;<br>902.2189	1246.3496;<br>902.2224	3;<br>4	-4.1;<br>-3.9	HCC_Hep_I
L15S7 + 4 DBA;<br>L15S7 + 3 DBA	1316.0475;<br>954.4960	1316.0524;<br>954.4996	3;<br>4	-3.6;<br>-3.7	HCC_Hep_I
L15S8 + 5 DBA;<br>L15S8 + 4 DBA	1385.7505;<br>1006.7722	1385.7553;<br>1006.7767	3;<br>4	-3.5;<br>-4.5	HCC_Hep_I
L15S9 + 6 DBA	1455.4531	1455.4582	3	-3.5	HCC_Hep_I
L15S6SA1 + 3 DBA	1343.3788	1343.3814	3	-1.9	HCC_Hep_I
L15S7SA1 + 4 DBA	1413.0771	1413.0842	3	-5.0	HCC_Hep_I
L15S8SA1 + 5 DBA	1482.7838	1482.7871	3	-2.2	HCC_Hep_I
L15S9SA1 + 6 DBA	1552.4829	1552.4900	3	-4.6	HCC_Hep_I
L16S5 + 2 DBA	1235.3196	1235.3241	3	-3.6	HCC_Hep_II
L16S6 + 3 DBA	1305.0220	1305.0269	3	-3.8	HCC_Hep_I
L17S7 + 4 DBA;	1442.4179	1442.4229	3	-3.5	HCC_Hep_I
L17S8 + 5 DBA;<br>L17S8 + 4 DBA	1512.1190;<br>1101.5546	1512.1258;<br>1101.5546	3;<br>4	-4.5;<br>-4.5	HCC_Hep_I;<br>CCD_Hep_I
L17S9 + 6 DBA;<br>L17S9 + 5 DBA	1581.8237;<br>1153.8259	1581.8287;<br>1153.8317	3;<br>4	-3.1;<br>-5.0	HCC_Hep_I;<br>CCD_Hep_I
L17S10 + 7 DBA;<br>L17S10 + 6 DBA	1651.5225;<br>1206.1019	1651.5315;<br>1206.1089	3;<br>4	-5.5;<br>-5.8	CCD_Hep_I
L17S8SA1 + 5 DBA	1609.1514	1609.1576	3	-3.9	HCC_Hep_I
L17S9SA1 + 6 DBA;<br>L17S9SA1 + 5 DBA	1678.8567;<br>1226.6016	1678.8605;<br>1226.6056	3;<br>4	-2.2;<br>-3.2	HCC_Hep_I
L19S7 + 3 DBA	1144.0529	1144.0553	4	-2.1	CCD_Hep_I
L19S8 + 4 DBA	1196.3250	1196.3325	4	-6.2	CCD_Hep_I
L19S9 + 6 DBA;<br>L19S9 + 5 DBA	1708.1957;<br>1248.6063	1708.1992;<br>1248.6096	3;<br>4	-2.0;<br>-2.6	HCC_Hep_I
L19S10 + 7 DBA;<br>L19S10 + 6 DBA	1777.8972;<br>1300.8829	1777.9020;<br>1300.8868	3;<br>4	-2.7;<br>-3.0	HCC_Hep_I;<br>CCD_Hep_I
L19S11 + 7 DBA	1353.1570	1353.1639	4	-5.1	CCD_Hep_I
L19S9SA1 + 5 DBA	1321.3777	1321.3835	4	-4.4	HCC_Hep_I
L19S10SA1 + 6 DBA	1373.6537	1373.6606	4	-5.0	HCC_Hep_I
L19S11SA1 + 7 DBA	1426.1795[2]	1426.1896[2]	4	-7.1	HCC_Hep_I
L21S8 + 4 DBA	1291.1119	1291.1103	4	1.2	CCD_Hep_I
L21S9 + 5 DBA	1343.3850	1343.3875	4	-1.8	CCD_Hep_I
L21S10 + 7 DBA;<br>L21S10 + 6 DBA	1904.2695;<br>1395.6591	1904.2725;<br>1395.6646	3;<br>4	-1.6;<br>-4.0	HCC_Hep_II;<br>HCC_Hep_I
L21S11 + 7 DBA	1447.9345	1447.9418	4	-5.0	HCC_Hep_I
L21S12 + 8 DBA	1500.2139	1500.2189	4	-3.3	CCD_Hep_I
L21S13 + 9 DBA	1552.4862	1552.4961	4	-6.4	CCD_Hep_I
L21S10SA1 + 6 DBA	1468.4393	1468.4385	4	0.6	HCC_Hep_I
L21S11SA1 + 7 DBA	1520.9661[2]	1520.9675[2]	4	-0.9	HCC_Hep_I
L23S10 + 6 DBA	1490.4340	1490.4425	4	-5.7	HCC_Hep_I
L23S11 + 7 DBA	1542.7180	1542.7197	4	-1.1	HCC_Hep_I
L23S12 + 8 DBA	1594.9962	1594.9968	4	-0.4	HCC_Hep_I
L23S13 + 9 DBA	1647.2701	1647.2740	4	-2.3	CCD_Hep_I
L23S14 + 10 DBA	1699.7952[2]	1699.8029[2]	4	-4.5	CCD_Hep_I
L23S11SA1 + 7 DBA	1615.7424[2]	1615.7453[2]	4	-1.8	HCC_Hep_I
L25S11 + 7 DBA	1637.7466[2]	1637.7494	4	-1.7	HCC_Hep_I
L25S12 + 8 DBA	1689.7693	1689.7747	4	-3.2	HCC_Hep_I
L25S13 + 9 DBA;	1742.2935[2];	1742.3037[2];	4;	-5.8;	HCC_Hep_I;

L25S13 + 8 DBA	1367.8031	1367.8111	5	-5.8	CCD_Hep_I
L25S14 + 9 DBA	1409.6270	1409.6328	5	-4.1	CCD_Hep_I
L25S15 + 10 DBA	1451.6526[2]	1451.6560[2]	5	-2.3	CCD_Hep_II
L27S12 + 8 DBA	1784.7994[2]	1784.8044[2]	4	-2.7	HCC_Hep_II
L27S13 + 9 DBA	1837.0832	1837.0815	4	0.9	HCC_Hep_I
L27S14 + 10 DBA	1889.3586	1889.3587	4	0.0	HCC_Hep_I
L29S14 + 10 DBA	1984.1283	1984.1366	4	-4.2	HCC_Hep_I

[1]Average monoisotopic *m/z* at the 50% relative abundance level of the chromatographic peak in the XIC.

[2]Second isotope. DBA, dibutylamine.

Table S2. Experimental and theoretical masses, and accuracy of precursor ions.

Glycan structure	Composition	Experimental mass*	Theoretical mass	z	Acc. (ppm)	Figure reference	Reference file; MS^2 scan no.
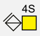	dp2S1	458.0589	458.0610	1	-4.6	Figure S3**b**	CCD_ABC_I; #4212<br>HCC_ABC_I; #4468<br>CCD_AC_I; #4388<br>HCC_AC_I; #4488<br>CCD_B_I; #4436^{2}<br>HCC_B_I; #4334
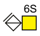	dp2S1	458.0589	458.0610	1	-4.6	Figure S3**c**	CCD_ABC_I; #4116<br>HCC_ABC_I; #4141<br>CCD_AC_I; #4180<br>HCC_AC_I; #4168^{2}
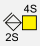	dp2S2	268.5055	268.5053	2	0.9	Figure 2**d**	CCD_ABC_I; #7094<br>CCD_AC_I; #6822<br>CCD_B_I; #6598<br>di-2S4S_std; #6568^{2}
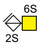	dp2S2	268.5056	268.5053	2	1.3	Figure 2**e**	CCD_ABC_I; #6470<br>HCC_ABC_I; #6678<br>CCD_AC_I; #6598<br>di-2S6S_std; #6082^{2}
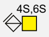	dp2S2	268.5054;<br>538.0179;<br>667.1699	268.5053;<br>538.0178;<br>667.1696	2;<br>1;<br>1	0.6;<br>0.2;<br>0.4	Figure S3**e–g**;<br>Figure S15**a–c**	HCC_ABC_I; #6810<br>HCC_AC_I; #6736<br>HCC_B_I; #6502<br>di-4S6S_std; #6490^{2}<br>di-4S6S_std; #6535^{2}<br>di-4S6S_std; #6513^{2}<br>HCC_B_CID; #6824<br>HCC_B_CID; #6827<br>HCC_B_CID; #6805
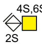	dp2S3	308.4831;<br>747.1249	308.4837;<br>747.1264	2<br>1	-1.8;<br>-2.0	Figure S3**h**	CCD_ABC_I; #9248<br>HCC_ABC_I; 9002<br>CCD_AC_I; #9610<br>CCD_B_I; #9194<br>HCC_B_I; #8532
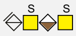	dp4S2	458.0593	458.0610	2	-3.7	Figure 2**f**<br>Figure S5**b,d**	CCD_AC_I; #8323<br>HCC_AC_I; #8327
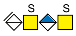	dp4S2	458.0593	458.0610	2	-3.7	Figure 2**g**<br>Figure S5**c,e**	CCD_B_I; #8435<br>HCC_B_I; #8109
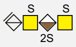	dp4S3	331.6896	331.6905	3	-2.7	Figure 2**i**<br>Figure S5**i**	CCD_AC_I; #10853
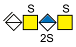	dp4S3	331.6895;<br>562.6145	331.6905;<br>562.6153	3<br>2	-3.0;<br>-1.4	Figure S5**k**	CCD_B_I; #10424<br>HCC_B_I; #9668
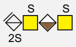	dp4S3	331.6896	331.6905	3	-2.7	Figure 2**h**<br>Figure S5**h**	CCD_AC_I; #10344
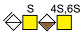	dp4S3	331.6897	331.6905	3	-2.4	Figure S5**j**	HCC_AC_I; #9743
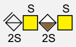	dp4S4	268.5044	268.5053	4	-3.2	Figure S6**l**	CCD_AC_I; #13182
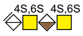	dp4S4	602.5930	602.5937	2	-1.2		HCC_AC_I; #11355
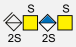	dp4S4	602.5916	602.5937	2	-3.5		CCD_B_I; #12589
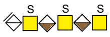	dp6S3	458.0595	458.0610	3	-3.3	Figure S5**m**	CCD_AC_I; #10930<br>HCC_AC_I; #10732
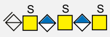	dp6S3	458.0595	458.0610	3	-3.3	Figure S5**n**	CCD_B_I; #11333<br>HCC_B_I; #10418
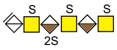	dp6S4	363.2820	363.2831	4	-3.1	Figure S5**o**	CCD_AC_I; #13368
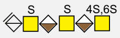	dp6S4	363.2823	363.2831	4	-2.3		HCC_AC_I; #11634
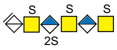	dp6S4	363.2820	363.2831	4	-3.1		CCD_B_I; #12795
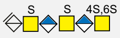	dp6S4	363.2825	363.2831	4	-1.7		HCC_B_I; #11144

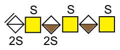	dp6S5	306.4156	306.4164	5	-2.6	Figure S5**p**	CCD_AC_I; #14702
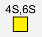	dp1S2	379.9951	379.9963	1	-3.2		CCD_ABC_I; #4586<br>HCC_ABC_I; #4538<br>CCD_AC_I; #4714<br>HCC_AC_I; #4683<br>CCD_B_I; #4698<br>HCC_B_I; #4529
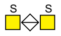	dp3S2	379.0491	379.0503	2	-3.0		CCD_AC_I; #6492<br>HCC_AC_I; #6419<br>CCD_B_I; #6339<br>HCC_B_I; #6205
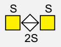	dp3S3	483.6034;<br>419.0276;<br>279.0158	483.6046;<br>419.0287;<br>279.0167	2<br>3	-2.4;<br>-2.6;<br>-3.1	Figure 3**a**<br>Figure S7**b**<br>Figure S15**d**	CCD_AC_I; #9367<br>HCC_AC_I; #8752<br>CCD_B_I; #8910<br>HCC_B_CID; #8798
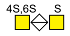	dp3S3	419.0278;<br>279.0162	419.0287;<br>279.0167	2<br>3	-2.0;<br>-1.7	Figure 3**b**<br>Figure S7**f**	HCC_AC_I; #8515<br>HCC_B_II; #8199[2]
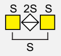	dp3S4	523.5812	523.5830	2	-3.3	Figure 3**c**<br>Figure S7**c**	CCD_AC_I; #11808<br>HCC_AC_I; #10756<br>CCD_B_I; #11111<br>HCC_B_I; #10289
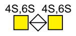	dp3S4	523.5819	523.5830	2	-2.0	Figure 3**d**<br>Figure S7**e**	HCC_AC_I; #10206<br>HCC_B_I; #10154
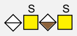	dp4S2	467.0644	467.0663	2	-4.1	Figure S7**g**	CCD_AC_I; #7804
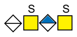	dp4S2	467.0650	467.0663	2	2.8	Figure S7**h**	CCD_B_I; #8342
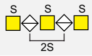	dp5S4	777.7121	777.7146	2	-3.2		CCD_AC_I; #12784<br>CCD_B_I; #12307
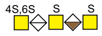	dp5S4	777.7123	777.7146	2	-3.0		CCD_AC_I; #12614<br>HCC_AC_I; #11047
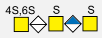	dp5S4	323.7770	323.7778	4	-2.3	Figure S7**i**	CCD_B_I; #11955<br>HCC_B_II; #10440[2]
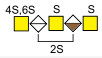	dp5S5	817.6903	817.6930	2	-3.3		CCD_AC_I; #13990<br>HCC_AC_I; #12106
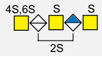	dp5S5	882.2652	882.2689	2	-4.2		CCD_B_I; #13494
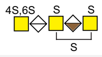	dp5S5	817.6913	817.6930	2	-2.1		HCC_AC_I; #11811
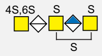	dp5S5	753.1156	753.1171	2	-2.0		HCC_B_I; #11257
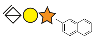	ΔL3S0	595.1650[1]	595.1668	1	-3.0	Figure S10**a**	CCD_AC_I; #16190[2]<br>HCC_AC_I; #13746
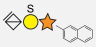	ΔL3S1	675.1214[1]	675.1236	1	-3.3	Figure S10**b**	CCD_AC_I; #17104[2]<br>HCC_AC_I; #14382
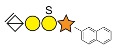	ΔL4S1	837.1744	837.1764	1	-2.4	Figure 4**a**<br>Figure S9**c**	HCC_AC_I; #14107
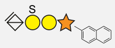	ΔL4S1	837.1738[1]	837.1764	1	-3.1	Figure 4**b**<br>Figure S9**d**	CCD_AC_I; #16852[2]<br>HCC_AC_I; #14248<br>CCD_B_I; #16260<br>HCC_B_I; #13566
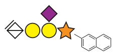	ΔL4S0SA1	523.6522[1]	523.6539	2	-3.2	Figure S11**c**	CCD_ABC_I; #13666<br>HCC_ABC_I; #13525<br>CCD_AC_I; #16341<br>HCC_AC_I; #13878[2]
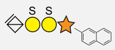	ΔL4S2	458.0623	458.0630	2	-1.4	Figure S12**a**	CCD_ABC_I; #14972<br>HCC_ABC_I; #14463<br>CCD_AC_I; #17478<br>HCC_AC_I; #14807[2]
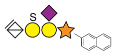	ΔL4S1SA1	563.6315[1]	563.6323	2	-1.3	Figure S11**e**	CCD_ABC_I; #14224<br>HCC_ABC_I; #14022<br>CCD_AC_I; #17111<br>HCC_AC_I; #14472[2]<br>CCD_B_I; #16528<br>HCC_B_I; #13793
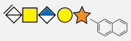	ΔL5S0	486.6342[1]	486.6355	2	-2.7	Figure S10**c**	CCD_ABC_I; #14200[2]<br>HCC_ABC_I; #13909
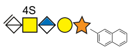	ΔL5S1	526.6122	526.6139	2	-3.2	Figure S10**d**	CCD_ABC_I; #15132

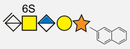	ΔL5S1	526.6128	526.6139	2	-2.1	Figure S10**e**	HCC_ABC_I; #14604
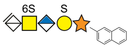	ΔL5S2	566.5897	566.5923	2	-4.6	Figure S10**f**	CCD_ABC_I; #15302[2]<br>HCC_ABC_I; #14803
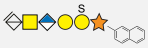	ΔL6S1	607.6392	607.6403	2	-1.8	Figure 4**c**<br>Figure S9**g**	CCD_ABC_I; #14179<br>HCC_ABC_I; #13955<br>CCD_AC_I; #16956<br>HCC_AC_I; #14336[2]
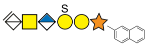	ΔL6S1	607.6390	607.6403	2	-2.1	Figure 4**d**<br>Figure S9**h**	CCD_ABC_I; #14195<br>CCD_AC_I; #17043[2]
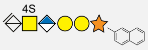	ΔL6S1	607.6383[1]	607.6403	2	-3.3	Figure S9**i**	CCD_ABC_I; #14691<br>CCD_AC_I; #17363<br>CCD_B_I; #16627[2]<br>HCC_B_I; #13965
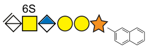	ΔL6S1	607.6381[1]	607.6403	2	-3.6	Figure S9**j**	CCD_ABC_I; #14243<br>HCC_ABC_I; #14067[2]<br>CCD_AC_I; #17203<br>HCC_AC_I; #14583
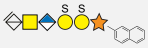	ΔL6S2	647.6168	647.6187	2	-2.9	Figure S12**b**	CCD_ABC_I; #15023<br>CCD_AC_I; #17583[2]
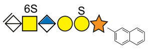	ΔL6S2	647.6167	647.6187	2	-3.1	Figure 4**e**<br>Figure S9**m**	HCC_AC_I; #14906
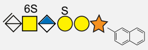	ΔL6S2	647.6170	647.6187	2	-2.6	Figure 4**f**<br>Figure S9**n**	CCD_ABC_I; #15203[2]<br>HCC_ABC_I; #14675<br>CCD_AC_I; #17747<br>HCC_AC_I; #14970<br>CCD_B_I; #17238<br>HCC_B_I; #14275
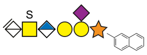	ΔL6S1SA1	501.7879[1]	501.7896	3	-3.3	Figure S11**d**	CCD_ABC_I; #14856<br>HCC_ABC_I; #14376[2]<br>HCC_AC_I; #14719<br>CCD_B_I; #16846<br>HCC_B_I; #14086
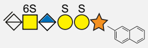	ΔL6S3	458.0604[1]	458.0623	3	-4.1	Figure S12**c**	HCC_ABC_I; #14847
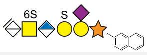	ΔL6S2SA1	528.4393[1]	528.4418	3	-4.8	Figure S11**f**	HCC_ABC_I; #14751
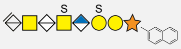	ΔL8S2	837.1707	837.1745	2	-4.5	Figure S13**a**	CCD_AC_I; #17817
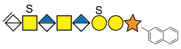	ΔL8S2	837.1707	837.1745	2	-4.5	Figure S13**b**	CCD_B_I; #17430
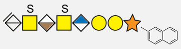	ΔL8S2	837.1711[1]	837.1745	2	-4.1	Figure S13**c**,**e**	CCD_AC_I; #17874-5[2]<br>HCC_AC_I; #15056-7
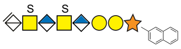	ΔL8S2	837.1710[1]	837.1745	2	-4.1	Figure S13**d**,**f**	CCD_B_I; #17570-1[2]<br>HCC_B_I; #14396-7
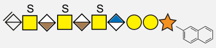	ΔL10S3	710.8009[1]	710.8033	3	-3.4	Figure S13**g**	CCD_AC_I; #17957<br>HCC_AC_I; #15091
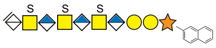	ΔL10S3	710.8002[1]	710.8033	3	-4.4	Figure S13**h**<br>Figure S16	CCD_B_I; #17778[2]<br>HCC_B_I; #14463<br>CCD_B_I; #17778-80,<br>CCD_B_II; #14999-15003[3]
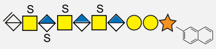	ΔL10S4	780.5023[1]	780.5062	3	-5.0	Figure S13**i**	CCD_B_I; #17880
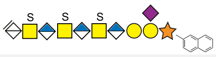	ΔL10S3SA1	807.8316[1]	807.8351	3	-4.3	Figure S11**g**	CCD_B_I; #17758<br>HCC_B_I; #14415[2]
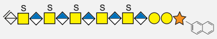	ΔL14S5	(1102.9441;<br>794.6688)<br>609.7037[1]	(1102.9500;<br>794.6728)<br>609.7064	(3;<br>4)<br>5	(-5.4;<br>-5.0)<br>-4.4	Figure S13**j**	(CCD_B_I; #17851;<br>17870;17861<br>HCC_B_I; #14466)<br>CCD_B_II; #15080[2]

*Depending on the spectrum scan number, the two last decimals differ slightly. This explains any differences between the presented decimal values in the tables and figures. Experimental monoisotopic masses were taken directly prior to the reference MS^2 scan number, except when it was taken [1]at the average monoisotopic *m/z* at the 50% relative abundance level of the chromatographic peak in the XIC.

**Where several reference files are shown, the experimental mass was taken from the first example or as indicated by [2].

Table S3. Annotations, experimental and theoretical masses, and accuracies of fragment ions included in Figures S5, S7, S8, and S10–S12.

Fragment ion number	Proposed structure	Experimental mass[1]	Theoretical mass	*z*	Accuracy (ppm)
1	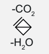	113.0245	113.0244	1	0.9
2	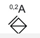	115.0038	115.0036	1	1.7
3	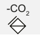	131.0350	131.0350	1	0.0
4	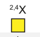	142.0508	142.0504	1	2.8
5	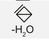	157.0140	157.0142	1	-1.3
6	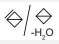	175.0244	175.0248	1	-2.3
7	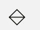	193.0349	193.0354	1	-2.6
8	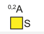	198.9914	198.9913	1	0.5
9	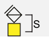	237.5316	237.5322	2	-2.3
10	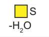	282.0282	282.0289	1	-2.5
11	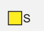	300.0386	300.0395	1	-3.0
12	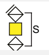	316.5420	316.5429	2	-2.8
13	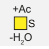	324.0387	324.0395	1	-2.5
14	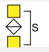	339.0710	339.0719	2	-2.5
15	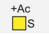	342.0494	342.0501	1	-2.0
16	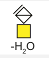	360.0939	360.0936	1	0.8
17	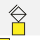	378.1034	378.1042	1	-2.1
18	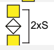	379.0491	379.0503	2	-3.0
19	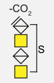	396.0829	396.0877	2	-12.1
20	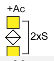	400.0543	400.0556	2	-3.1
21	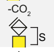	414.0701	414.0712	1	-2.7
22	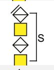	418.0815	418.0826	2	-2.6
23	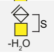	440.0494	440.0504	1	-2.3
24	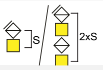	458.0598	458.0610	1; 2	-2.6
25	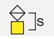	476.0699	476.0716	1	-3.6
26	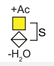	500.0702	500.0716	1	-2.8
27	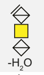	536.1245	536.1257	1	-2.2
28	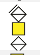	554.1375	554.1363	1	2.2
29	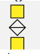	599.1922	599.1942	1	-3.3
30	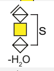	618.0813	618.0825	1	-1.9
31	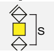	634.0925	634.0931	1	-0.9
32	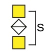	679.1481	679.1510	1	-4.3
33	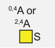	138.9707	138.9701	1	3.6

34	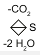	174.9705	174.9706	1	-0.6
35	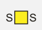	189.4942	189.4945	2	-1.6
36	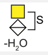	228.5264	228.5269	2	-2.0
37	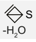	236.9707	236.9710	1	-1.3
38	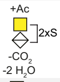	237.5042	237.5051	2	-3.6
39	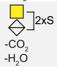	246.5093	246.5104	2	-4.3
40	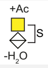	249.5314	249.5322	2	-3.0
41	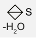	254.9813	254.9816	2	-1.2
42	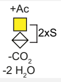	258.5097	258.5104	2	-2.5
43	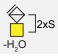	259.4987	259.5000	2	-4.8
44	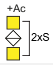	266.3672	266.3679	2	-2.8
45	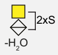	268.5046	268.5053	2	-2.4
46	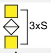	279.0161	279.0167	3	-2.0
47	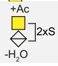	289.5099	289.5106	2	-2.2
48	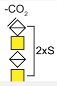	290.3741	290.3750	3	-3.0
49	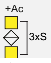	293.0196	293.0202	3	-2.0
50	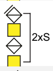	305.0375	305.0382	3	-2.4
51	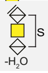	307.5369	307.5376	2	-2.3
52	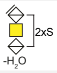	347.5152	347.5160	2	-2.3
53	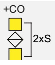	393.0468	393.0477	2	-2.3
54	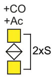	414.0527	414.0530	2	-0.7
55	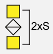	252.3636	252.3644	3	-3.2
56	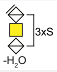	257.9931	257.9938	3	-2.8
57	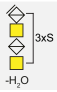	325.6865	325.6870	3	-1.4
58	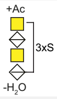	345.6931	345.6940	3	-2.7
59	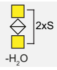	370.0439	370.0450	2	-2.8
60	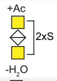	391.0439	391.0503	2	-2.4
61	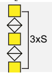	405.3859	405.3872	3	-3.1

62	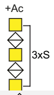	419.3896	419.3907	3	-2.6
63	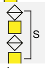	429.0870	427.0879	2	-2.1
64	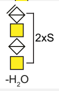	449.0546	449.0557	2	-2.4
65	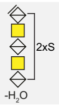	537.0706	537.0718	2	-2.1
66	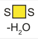	180.4889	180.4892	2	-1.7
67	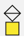	396.1130	396.1148	1	-4.5
1’	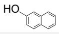	143.0501	143.0502	1	-0.7
2’	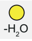	161.0453	161.0455	1	-1.2
3’	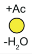	203.0558	203.0561	1	-1.5
4’	n.d.	227.0557	-	1	-
5’	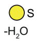	241.0018	241.0023	1	-2.1
6’	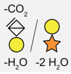	275.0765	275.0772	1	-2.5
7’	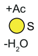	283.0121	283.0129	1	-2.8
8’	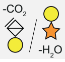	293.0871	293.0878	1	-2.4
9’	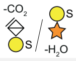	373.0437	373.0446	1	-2.4
10’	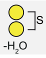	403.0543	403.0551	1	-2.0
11’	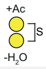	445.0647	445.0657	1	-2.2
12’	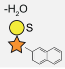	499.0897	499.0915	1	-3.6
13’	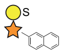	517.1003	517.1021	1	-3.5
14’	n.d.	521.1508	-	1	-
15’	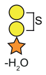	535.0956	535.0974	1	-3.4
16’	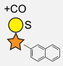	545.0957	545.0970	1	2.4
17’	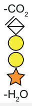	569.1713	569.1723	1	-1.8
18’	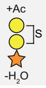	577.1064	577.1080	1	-2.8
19’	n.d.	645.1481	-	1	-
20’	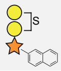	679.1532	679.1549	1	-2.5
21’	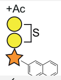	721.1636	721.1655	1	-2.6
22’	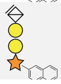	757.2177	757.2196	1	-2.5

23’	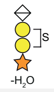	355.0609	355.0611	2	-0.6
24’	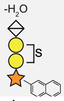	418.0836	418.0846	2	-2.3
25’	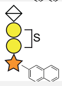	427.0887	427.0899	2	-2.7
26’	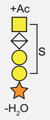	477.6041	477.6061	2	-4.2
27’	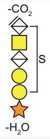	513.6155	513.6167	2	-2.2
28’	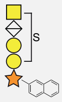	528.6277	528.6296	2	-3.5
29’	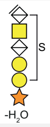	535.6098	535.6116	2	-3.3
30’	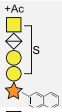	549.6331	549.6349	2	-3.2
31’	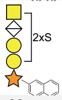	568.6072	568.6080	2	-1.3
32’	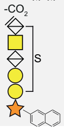	585.6440	585.6454	2	-2.4
33’	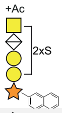	589.6111	589.6133	2	-3.6
34’	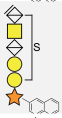	607.6385	607.6403	2	-3.0
35’	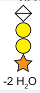	613.1601	613.1621	1	-3.3
36’	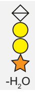	631.1716	631.1727	1	-1.7
37’	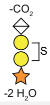	649.1279	649.1291	1	-1.8
38’	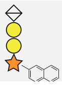	775.2277	775.2302	1	-3.2
39’	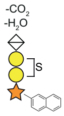	793.1840	793.1866	1	-3.3

40’	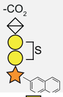	811.1924	811.1972	1	-5.9
41’	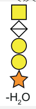	834.2484	834.2521	1	-4.4
42’	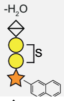	837.1762	837.1764	1	-0.2
43’	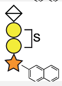	855.1849	855.1870	1	-2.5
44’	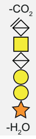	948.2844	948.2838	1	0.6
45’	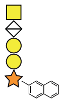	978.3064	978.3096	1	-3.3
46’	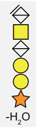	992.2693	992.2736	1	-4.3
47’	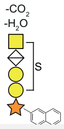	996.2617	996.2660	1	-4.3
48’	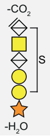	1028.2368	1028.2406	1	-3.7
49’	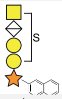	1058.2676	1058.2664	1	1.1
50’	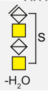	409.0763	409.0773	2	-2.4
51’	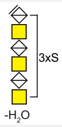	452.0558	452.0575	3	-3.7
52’	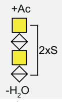	479.0649	479.0663	2	-2.9
53’	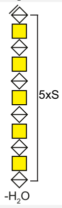	489.6646	489.6653	5	-1.4
54’	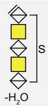	497.0916	497.0934	2	-3.5

55’	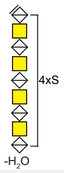	497.5638	497.5664	4	-5.2
56’	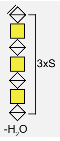	510.7334	510.7348	3	-2.8
57’	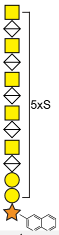	578.1015	578.1021	5	-1.0
58’	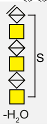	598.6310	598.6331	2	-3.4
59’	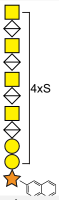	608.1207	608.1124	4	13.7
60’	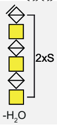	638.6094	638.6115	2	-3.2
61’	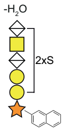 / 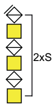	647.6149	647.6187/ 647.6168	2	-5.9/ -2.9
62’	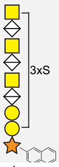	658.1274	658.1295	3	-3.1
63’	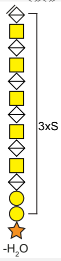	686.3910	686.3920	4	-1.5
64’	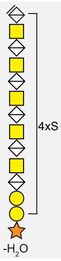	706.3796	706.3812	4	-2.3

65’	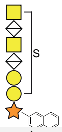	718.1824	718.1853	2	-4.0
66’	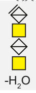	739.2023	739.2051	1	-3.8
67’	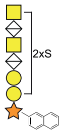	758.1589	758.1637	2	-6.3
68’	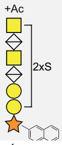	779.1656	779.1690	2	-4.4
69’	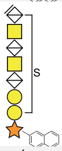	797.1924	797.1961	2	-4.6
70’	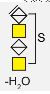	819.1591	819.1619	1	-3.4
71’	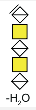	915.2332	915.2372	1	-4.4
72’	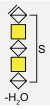	995.1892	995.1940	1	-4.8
73’	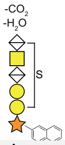	1172.2941	1172.2981	1	-3.4
74’	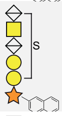	1234.2942	1234.2985	1	-3.5
75’	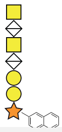	1357.4183	1357.4211	1	-2.1
1”	n.d.	359.0973	-	1	-
2”	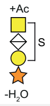	396.5781	396.5797	2	-4.0
3”	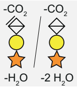	407.1180	407.1195	1	-3.7
4”	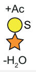	415.0536	415.0552	1	-3.9
5”	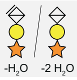	451.1069	451.1093	1	-5.3
6”	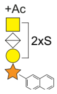	508.5852	508.5869	2	-3.2

7”	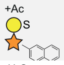	559.1105	559.1127	1	-3.9
8”	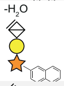	577.1542	577.1562	1	-3.5
9”	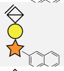	595.1647	595.1668	1	-3.5
10”	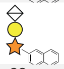	613.1774	613.1774	1	0.0
11”	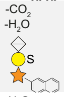	631.1315	631.1338	1	-3.6
12”	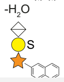	675.1208	675.1236	1	-4.1
13”	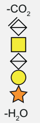	786.2280	786.2310	1	-3.8
14”	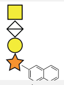	816.2531	816.2568	1	-4.5
15”	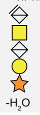	830.2177	830.2208	1	-3.7
1°	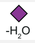	290.0877	290.0881	1	-1.4
2°	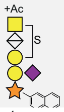	463.1182	463.1193	3	-2.3
3°	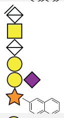	475.1357	475.1373	3	-3.4
4°	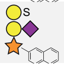	484.6204	484.6215	2	-2.3
5°	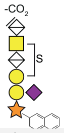	487.1245	487.1263	3	-3.7
6°	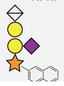	532.6571	532.6592	2	-3.8
7°	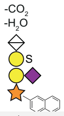	541.6357	541.6374	2	-3.0
8°	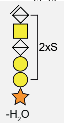	575.5887	575.5900	2	-2.2
9°	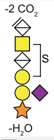	637.1674	637.1695	2	-3.2
10°	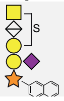	674.1745	674.1773	2	-4.1
11°	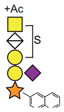	695.1802	695.1826	2	-3.4

12°	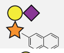	728.2362	728.2407	1	-6.2
13°	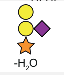	746.2347	746.2360	1	-1.7
14°	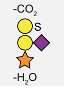	782.2007	782.2030	1	-2.9
15°	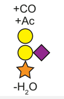	816.2407	816.2415	1	-1.0
16°	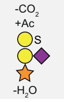	824.2101	824.2136	1	-4.2
17°	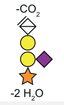	842.2560	842.2571	1	-1.3
18°	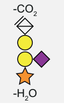	860.2659	860.2677	1	-2.1
19°	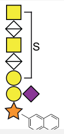	863.7302	863.7330	2	-3.2
20°	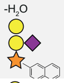	872.2807	872.2829	1	-2.5
21°	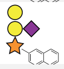	890.2916	890.2935	1	-2.1
22°	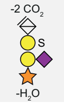	896.2314	896.2347	1	-3.7
23°	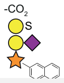	926.2601	926.2605	1	-0.4
24°	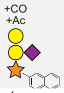	960.2969	960.2990	1	-2.2
25°	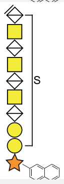	986.7482	986.7518	2	-3.6
26°	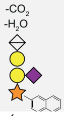	1004.3209	1004.3252	1	-4.3
27°	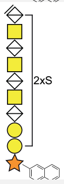	1026.7256	1026.7302	2	-4.5
1*	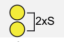	250.0070	250.0076	2	-2.4
2*	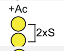	271.0120	271.0129	2	-3.3
3*	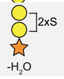	307.0228	307.0235	2	-2.1
4*	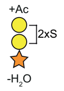	328.0279	328.0288	2	-2.6

5*	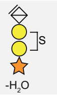	346.0558	346.0558	2	0.0
6*	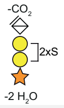	355.0332	355.0340	2	-2.3
7*	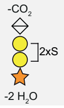	364.0385	364.0393	2	-2.2
8*	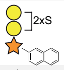	379.0513	379.0522	2	-2.4
9*	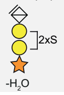	386.0332	386.0342	2	-2.6
10*	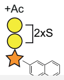	400.0565	400.0575	2	-2.5
11*	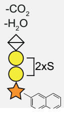	436.0666	436.0681	2	-3.3
12*	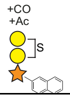	749.1580	749.1580	1	-3.2

[1]Examples from spectra.

n.d., not determined.

References

1 Kailemia, M. J. *et al.* Differentiating chondroitin sulfate glycosaminoglycans using collision-induced dissociation; uronic acid cross-ring diagnostic fragments in a single stage of tandem mass spectrometry. *Eur. J. Mass Spectrom.* **21**, 275-285, doi:10.1255/ejms.1366 (2015).

2 Wheeler, S. F. & Harvey, D. J. Negative ion mass spectrometry of sialylated carbohydrates: discrimination of N-acetylneuraminic acid linkages by MALDI-TOF and ESI-TOF mass spectrometry. *Anal. Chem.* **72**, 5027-5039, doi:10.1021/ac000436x (2000).
